# SAveRUNNER: an R-based tool for drug repurposing

**DOI:** 10.1186/s12859-021-04076-w

**Published:** 2021-03-23

**Authors:** Giulia Fiscon, Paola Paci

**Affiliations:** 1 Institute for Systems Analysis and Computer Science, Antonio Ruberti”, National Research Council, Rome, Italy; 2 Fondazione Per La Medicina Personalizzata, Via Goffredo Mameli, 3/1, Genoa, Italy; 3 Department of Computer, Control and Management Engineering, Sapienza University of Rome, Rome, Italy

**Keywords:** Drug repurposing, Network theory, Network medicine

## Abstract

**Background:**

Currently, no proven effective drugs for the novel coronavirus disease COVID-19 exist and despite widespread vaccination campaigns, we are far short from herd immunity. The number of people who are still vulnerable to the virus is too high to hamper new outbreaks, leading a compelling need to find new therapeutic options devoted to combat SARS-CoV-2 infection. Drug repurposing represents an effective drug discovery strategy from existing drugs that could shorten the time and reduce the cost compared to de novo drug discovery.

**Results:**

We developed a network-based tool for drug repurposing provided as a freely available R-code, called SAveRUNNER (Searching off-lAbel dRUg aNd NEtwoRk), with the aim to offer a promising framework to efficiently detect putative novel indications for currently marketed drugs against diseases of interest. SAveRUNNER predicts drug–disease associations by quantifying the interplay between the drug targets and the disease-associated proteins in the human interactome through the computation of a novel network-based similarity measure, which prioritizes associations between drugs and diseases located in the same network neighborhoods.

**Conclusions:**

The algorithm was successfully applied to predict off-label drugs to be repositioned against the new human coronavirus (2019-nCoV/SARS-CoV-2), and it achieved a high accuracy in the identification of well-known drug indications, thus revealing itself as a powerful tool to rapidly detect potential novel medical indications for various drugs that are worth of further investigation. SAveRUNNER source code is freely available at https://github.com/giuliafiscon/SAveRUNNER.git, along with a comprehensive user guide.

**Supplementary Information:**

The online version contains supplementary material available at 10.1186/s12859-021-04076-w.

## Background

Currently, we are still facing a global pandemic caused by the new coronavirus SARS-CoV-2. The viral waves in Europe and the United States restarted in August and mid-September are driving the steep upward trend of the global daily tally for new COVID-19 cases that increases up to new high records, suggesting that this wave will be worse than the one that swept the countries over the spring–summer.

Even though mass vaccination campaigns have been started across countries, it remains difficult to achieve herd immunity in a short time and the proportion of the population that is susceptible to the new coronavirus is still insufficient for new outbreaks to peter out. To meet the compelling need of finding new therapeutic options devoted to combat SARS-CoV-2 infection [1, 2], promising insights come from drug repurposing, a recent strategy for identifying novel uses for drugs approved by the US Food and Drug Administration (FDA) outside the scope of their original medical indication [3]. Establishing whether an ‘old drug’ can be reused for new therapeutic purposes represents a faster and cheaper alternative to de novo drug discovery, which generally takes 2–3 billion dollars and 12–15 years [3]. Hence, drug repurposing strategy appears as a powerful solution for emerging diseases, such as COVID-19 [4]. In this context, we developed SAveRUNNER (Searching off-lAbel dRUg aNd NEtwoRk), a new network-based tool for drug repurposing that exploits concepts from the emerging field of network medicine [5–9]. According to the new paradigm of Network Medicine, diseases can be interpreted as local perturbations in the human interactome map (i.e., the cellular network of all physical molecular interactions), where the molecular determinants of a given disease (*disease genes*) are not randomly scattered, but co-localize and agglomerate in specific regions (*disease modules*) [6, 10]. Perturbations in these disease modules can contribute to pathobiological phenotype manifestation. From this perspective, also the drugs action can be interpreted as a local perturbation of the interactome and thus, for a drug to be on-target effective against a specific disease or to cause off-target adverse effects, its target proteins should be within or in the immediate vicinity of the corresponding disease module [11–13]. Inspired by this philosophy, SAveRUNNER predicts drug–disease associations by quantifying the vicinity between the drug targets and the disease-associated proteins in the human interactome via a novel network-based similarity measure that rewards associations between drugs and diseases located in the same network neighborhoods. SAveRUNNER yielded a high accuracy in the identification of well-known drug indications, as well as being able to provide interesting clues regarding off-label prediction of drugs to be repositioned against the new human coronavirus [14].

## Implementation

**Fig. 1:**
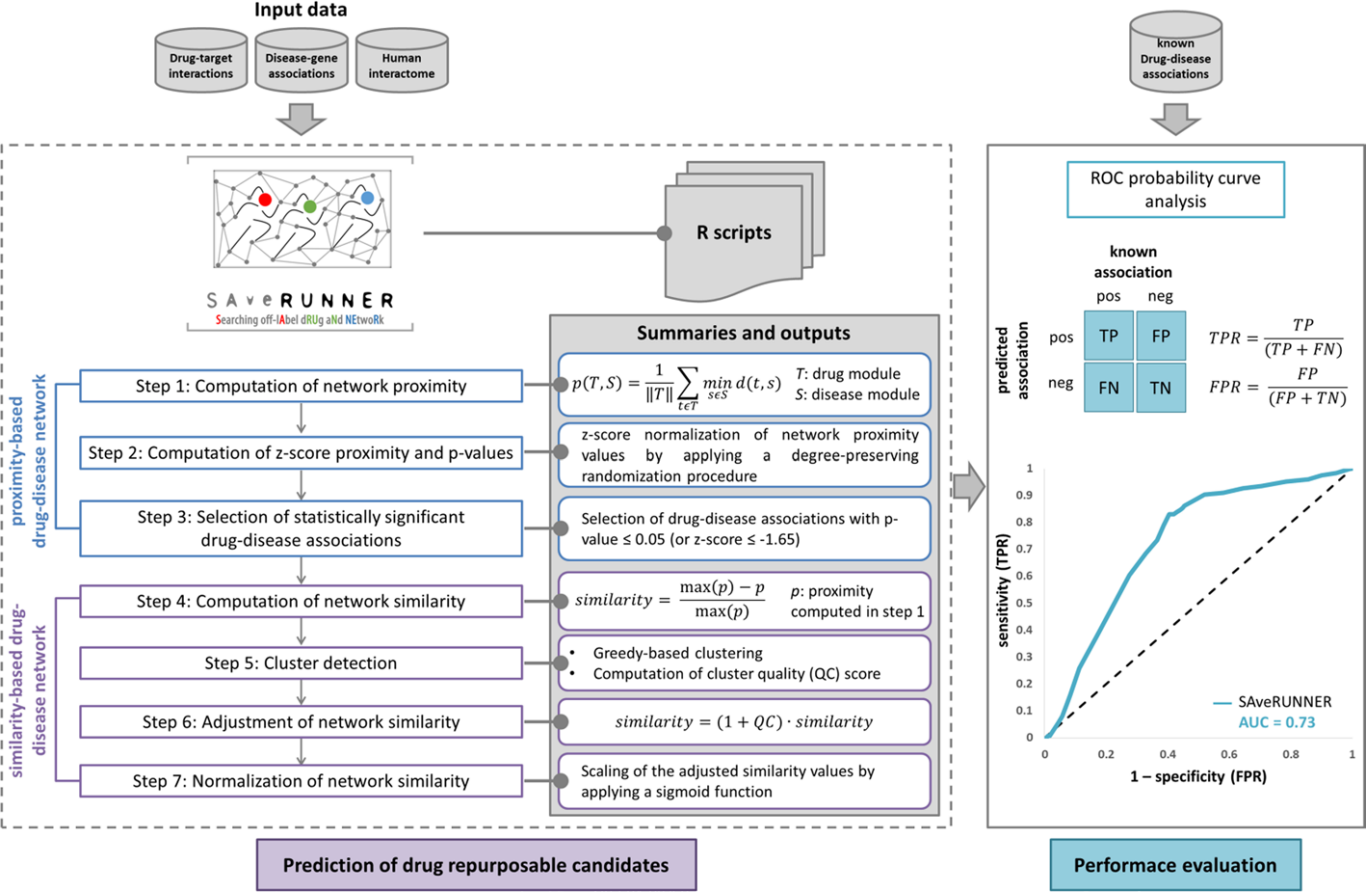
SAveRUNNER conceptual organization. SAveRUNNER takes as input a list of drug-target interactions and disease-gene associations, and releases as output predicted drug-disease associations by performing seven steps (dashed box of this flowchart). In particular, Steps 1–3 bring to the construction of a proximity-based bipartite drug-disease network, where nodes are both drugs and diseases, edges are the statistically significant drug-disease associations (*p *value $\le 0.05$, or z-score $\le -1.65$), weighted according to the proximity values; Steps 4–7 bring to the construction of a similarity-based bipartite drug-disease network, where the weights represent the adjusted similarity measure computed to prioritize the predicted drug-disease associations by rewarding the associations between drugs and diseases belonging to the same network neighborhood. Finally, the drug-disease associations predicted by SAveRUNNER were evaluated by performing a ROC curve probability analysis (solid line box of this flowchart). The ROC curve is computed for SAveRUNNER algorithm by plotting the true positive rate (TPR) placed on Y-axis against the false positive rate (FPR) placed on X-axis at various threshold settings. Diagonal grey line represents the line of no-discrimination between positive class (known drug-disease associations) and negative class (unknown drug-disease associations)

SAveRUNNER (Searching off-lAbel dRUg aNd NEtwoRk) is a network-based algorithm for drug repurposing that, taking as input a list of drug targets and disease genes, predicts drug-disease associations by computing a new network-based similarity measure to prioritize associations between drugs and diseases located in the same network neighborhoods by performing the following steps (Fig. 1).

### Computation of network proximity

SAveRUNNER implements the network-based proximity measure (Eq. 1) to investigate the extent to which disease and drug modules are close in the human interactome [11]:

1 $$p\left(T,S\right)=\frac{1}{\Vert T\Vert }\sum_{t\epsilon T}\underset{s\epsilon S}{\mathit{min}}d(t,s)$$

which is the average shortest path length between drug targets $t$ in the drug module $T$ and the nearest disease genes $s$ in the disease module $S$.

### Computation of z-score proximity and *p* values

The proximity value between a drug module T and a disease module S was z-score normalized by applying a degree-preserving randomization procedure. In particular, to compute the z-scores and the corresponding *p *values, SAveRUNNER builds a reference distance distribution corresponding to the expected distance between two randomly selected groups of proteins with the same size and degree distribution of the original sets of disease proteins and drug targets in the human interactome. This procedure is repeated 1,000 times and the z statistic, together with its *p *value, is computed by using the mean and the standard deviation of the reference distance distribution. A *p *value $\le$ 0.05 (corresponding to a z $\le$-1.65) was expected for a drug and disease module more proximal than expected by chance.

### Selection of statistically significant drug-disease associations

In order to filter out statistically insignificant drug-disease associations, a significance level for the *p *values should be set (typically, *p *value $\le$ 0.05). It means that, given a disease *A* and a drug *b*, if the *p *value associated to their distance in the human interactome is smaller of the chosen significance level, the probability that the off-label drug *b* would be effective for this disease *A* is greater than expected by chance.

### Computation of network similarity

The network proximity measure *p* defined in Eq. 1 is translated in a similarity measure (Eq. 2) assuming values in the range [0–1]:

2 $$similarity= \frac{\mathrm{max}\left(p\right)-p}{\mathrm{max}(p)}$$

Null similarity means that the corresponding disease and drug modules are very distal in the human interactome (i.e., $p$ is maximum); whereas maximum similarity means that the corresponding disease and drug modules are very proximal in the human interactome (i.e., $p$ equal to zero).

### Cluster detection

SAveRUNNER exploits a clustering algorithm based on greedy optimization of the network modularity [15] to detect groups of drugs and diseases in such a way that members in the same group (cluster) are more similar to each other than to those in other groups (clusters). The quality of each cluster is evaluated by SAveRUNNER through the computation of the quality cluster ($$QC)$$ score (Eq. 3):

3 $$QC=\frac{{W}_{in}}{{W}_{in}+ {W}_{out}+P}$$

where ${W}_{in}$ denotes the total weight of edges within the cluster, ${W}_{out}$ denotes the total weight of edges connecting this cluster to the rest of network, and $P$ is a penalty term which considers the node density within the cluster (i.e., the ratio of network nodes within each cluster).

### Adjustment of network similarity

SAveRUNNER uses the *QC* score to reward associations between drugs and diseases belonging to the same cluster, based on the assumption that if a drug and a disease group together is more likely that the drug can be effectively repurposed for that disease. Thus, drug and the disease that are members of the same cluster tend to be “more similar” and this translates into the adjustment for the similarity (Eq. 4):

4 $$similarity=(1+QC)\bullet similarity$$

In this way, if two nodes fall in the same cluster their similarity value increases by a factor proportional to the $QC$ score of the cluster which they belong; otherwise whether two nodes do not fall in the same cluster $QC$ is set to zero and their similarity value does not change.

### Normalization of network similarity

The similarity measure defined in Eq. 4 was normalized by applying the following sigmoid function (Eq. 5):

5 $$f(x)= \frac{1}{1+{e}^{-c(x-d)}}$$

where $x$ is the adjusted similarity measure (Eq. 4), *d* is the sigmoid midpoint (i.e., the value at which the function approaches to 0.5), *c* is the sigmoid steepness.

Eventually, SAveRUNNER releases a list of predicted/prioritized associations between drugs and diseases as a weighted bipartite drug-disease network, in which one set of nodes corresponds to drugs and the other one corresponds to diseases. A link between a drug and a disease occurs if the corresponding drug targets and disease genes are nearby in the interactome more than expected by chance (*p *value $\le$ 0.05) and the weight of their interaction corresponds to the adjusted and normalized similarity value.

## Results

### SAveRUNNER predictions of repurposable drugs in relation to COVID-19

**Fig. 2:**
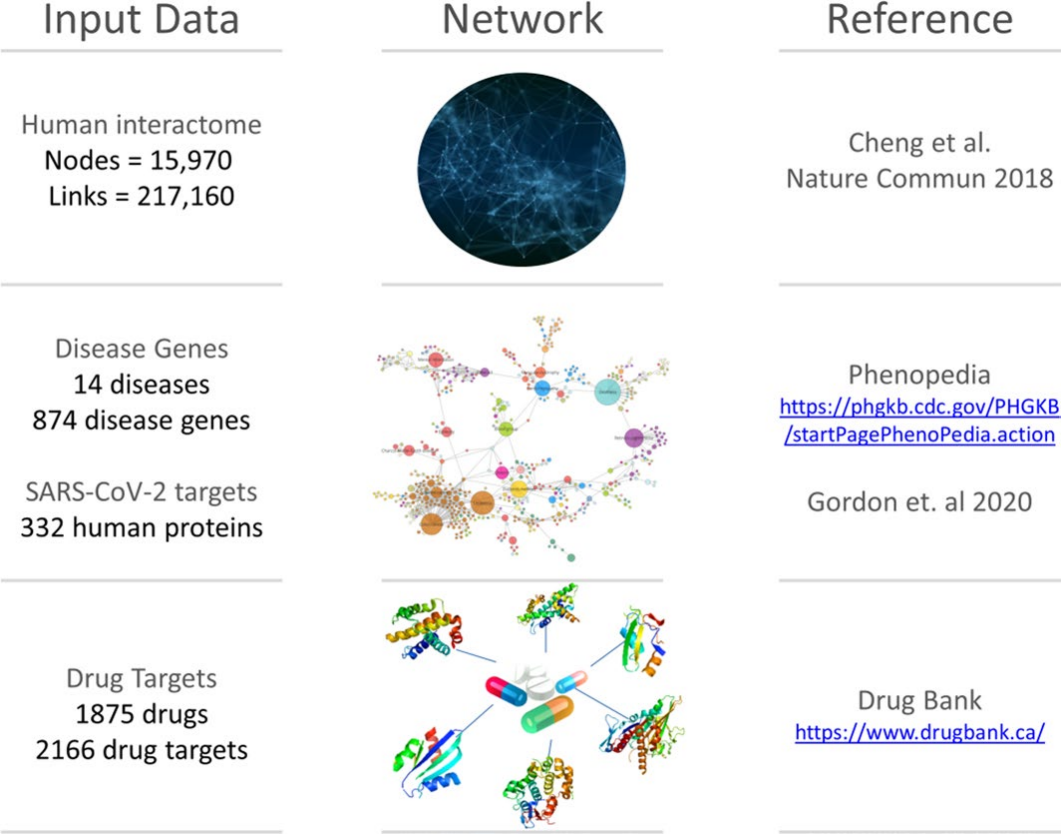
Used data resources. Summary of the all input data collection with the corresponding links to retrieve them

In order to evaluate the effectiveness of predicted drug–disease associations, we applied SAveRUNNER on several human diseases for which original medical indications were available. In particular, given the deep impact of the ongoing COVID-19 pandemic, we selected a panel of 15 disorders, including COVID-19 and 14 diseases related to COVID-19 (SARS-CoV-2) for genetic similarity, comorbidity, or for their association to drugs with ongoing clinical trials for treating COVID-19 patients. We tested Severe Acute Respiratory Syndrome (SARS) since it is caused by the coronavirus with the highest sequence identity with SARS-CoV-2 [12, 16] and there exists a well-established knowledge of its associated disease genes [17]. Moreover, we included also diabetes, cardiovascular diseases, and hypertension, whose comorbidity in COVID-19 patients is well documented [18, 19]; and finally other viral infections (i.e., malaria, HIV and Ebola) and immune disorders (i.e., rheumatoid arthritis), since drugs approved for their treatment are being investigated for their potential effect to fight coronavirus disease [1, 20–27]. COVID-19-associated genes were download from [28], where the authors identified 332 human proteins interacting with 26 SARS-CoV-2 proteins by using affinity purification mass spectrometry. Although this study has been carried out on human HEK293T kidney cells that do not represent the primary physiological site of infection, the authors verified that these proteins were preferentially highly expressed in lung tissue (the typical environment where the virus causes a major damage). Yet, the disease-associated genes for the other selected 14 diseases were downloaded from Phenopedia [17], the drug-target interactions were acquired from DrugBank [29], and the human interactome was retrieved from [11] (Fig. 2 and Additional file 1).

The list of network-predicted drugs potentially able to treat SARS-CoV-2 infection contains a total of 98 drugs (Additional file 2), including 54 (i.e., 55%) COVID-19 specific and 44 (i.e., 45%) in common with the candidate repurposable drugs found for SARS-CoV. The results of this study are broadly discussed in an our recent publication [14]. In Additional file 3, the data analysis details step-by-step were reported via a working example of SAveRUNNER application to COVID-19, from data collection, network, association analysis and predicted repurposable drugs.

### SAveRUNNER performance evaluation

The drug–disease associations predicted by the SAveRUNNER was then evaluated in terms of the Receiver Operating Characteristic (ROC) probability curve analysis (Fig. 1). The predicted associations were ranked according to increasing *p *values and a “real association” was assigned according to the well-known drug-disease associations downloaded from Therapeutic Target Database (TTD) [30]: 1 corresponds to predicted drug-disease association that is already known, 0 otherwise. For a specified *p *value threshold, the true positive rate (i.e., sensitivity) was calculated as the fraction of known associations that are correctly predicted, while the false positive rate (i.e., 1-specificity) was computed as the fraction of unknown associations that are predicted. The ROC probability curve was drawn based on these measures at different thresholds and the corresponding Area Under the Curve (AUC) was computed. The higher the AUC, the better the algorithm is at distinguishing between two classes (i.e., known drug-disease associations vs. unknown drug-disease associations). SAveRUNNER achieved over 70% accuracy (AUC = 0.73) for identifying well-known drug-disease relationships (Fig. 1), meaning that there is 73% chance that the SAveRUNNER algorithm will be able to distinguish between positive class (known drug-disease associations) and negative class (unknown drug-disease associations).

### Comparison with other methods

Among network-based methods proposed to predict direct drug–disease associations for drug repositioning [3, 31–35], the MBiRW algorithm has been shown to outperform other well-known network-based prediction methods [36–38] in correctly predicting true drug–disease associations. MBiRW adopts an effective mechanism to measure similarity for drugs and diseases and applies a Bi-Random walk (BiRW) algorithm to predict potential new indications for existing drugs [35]. These captivating results prompted us to implement a BiRW-based algorithm against which we compared the performance of SAveRUNNER. Details of BiRW-based algorithm implementation are provided as Additional file 4.

**Fig. 3:**
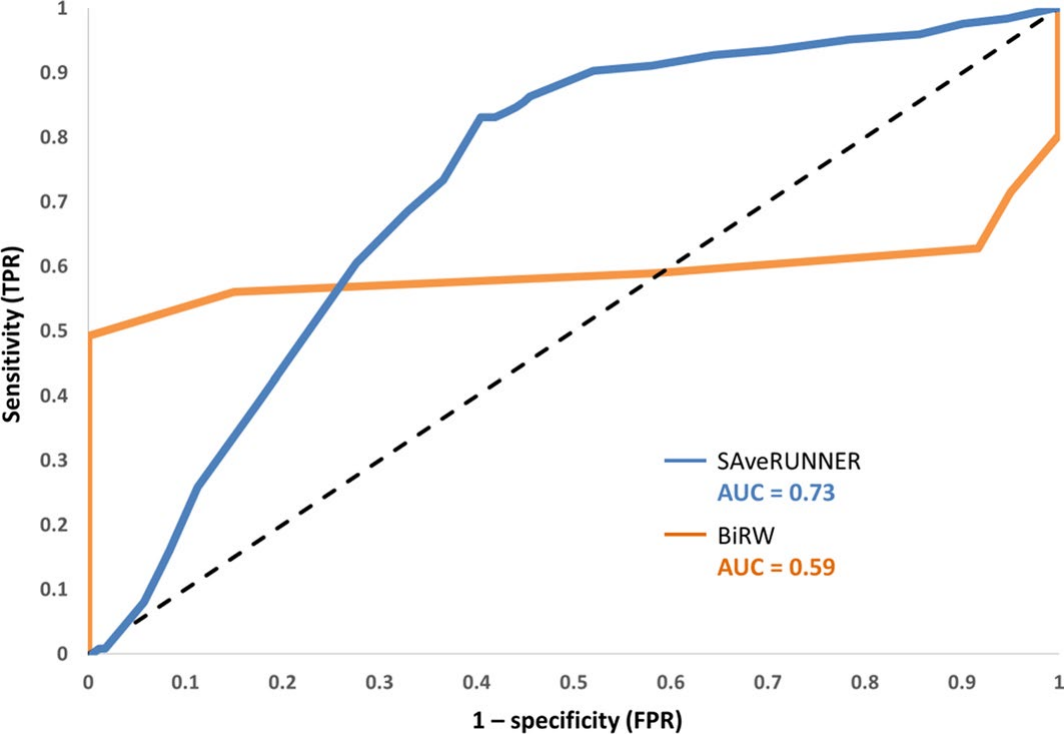
ROC curves for predicting drug–disease associations. The ROC curve is computed for SAveRUNNER algorithm (light blue curve) and BiRW algorithm (orange curve) by plotting the true positive rate (TPR), i.e., sensitivity placed on Y-axis against the false positive rate (FPR), i.e., 1-specificity placed on X-axis at various threshold settings. Diagonal grey line represents the line of no-discrimination between positive class (known drug-disease associations) and negative class (unknown drug-disease associations)

We evaluated and compared the drug–disease predictions provided by BiRW and SAveRUNNER in terms of ROC probability curves with their corresponding AUC. In particular, we found that SAveRUNNER yielded over 70% accuracy (AUC = 0.73) for identifying well-known drug-disease relationships and overcame the one obtained by the BiRW-based algorithm (AUC = 0.59). In other words, there is 73% chance that SAveRUNNER algorithm will be able to distinguish between known and unknown drug-disease associations against the 59% of the BiRW-based algorithm (Fig. 3).

To further evaluate the performances of SAveRUNNER, we compared its outcomes with the predictions obtained by a recent study, where the authors integrated several network-based drug repurposing strategies to prioritize 81 promising repurposing candidates against COVID-19 [13]. In particular, they combined three predictive approaches: (1) proximity-based methods that allowed to measure the distance between the viral protein targets and both (i) the targets of approved drugs and (ii) the differentially expressed genes induced by each drug; (2) diffusion-based methods to rank drugs based on the network similarity of their targets to COVID-19 protein targets; (3) machine learning methods relying on artificial intelligence network. These pipelines offered altogether twelve ranked lists that were merged using a rank aggregation algorithm in order to obtain a final list of 81 prioritized repurposable drugs. The overlap between these 81 repurposable drugs and the 98 ones predicted by SAveRUNNER is of 5 drugs, i.e. *isoniazid, lopinavir, romidepsin, sulfinpyrazone, tadalafil.*

However, although the use of more methodologies can provide more reliable and feasible drug repurposable candidates, the lack of a unified pipeline makes it difficult for non-expert users to exploit this approach for own research purposes.

## Availability and requirements

- *Project name*: SAveRUNNER.
- *Project page*: https://github.com/giuliafiscon/SAveRUNNER.git.
- *Operating system(s)*: macOS High Sierra 10.13.6, Windows 10 Pro.
- *Programming language*: R.
- *Other requirements*: R version 3.5.1 or higher.
- *License*: GNU AFFERO GENERAL PUBLIC LICENSE.

## Supplementary Information

**Additional file 1.** Table composed of three separate sheets. The first sheet reports the analyzed diseases with the corresponding number of disease-causing genes. The second sheet reports the analyzed FDA-approved drugs with the corresponding number of target proteins. The third sheet reports the analyzed diseases with the corresponding number of repurposable drugs predicted by SAveRUNNER.

**Additional file 2.** List of repurposable drugs for COVID-19 predicted by SAveRUNNER along with their statistics.

**Additional file 3.** Working example of SAveRUNNER application to COVID-19.

**Additional file 4.** Implementation of BiRW-based algorithm for drug repurposing.

## Data Availability

All data generated or analyzed during this study are included in this published article. SAveRUNNER code is open-source and it is available at https://github.com/giuliafiscon/SAveRUNNER.git, together with an exhaustive and well-documented user guide, which includes a detailed description of all R scripts, all input/output files through a working example of SAveRUNNER application on 15 diseases, including COVID-19.

## Abbreviations

FDA: Food and drug administration
SAveRUNNER: Searching off-lAbel dRUg aNd NEtwoRk
QC: Quality score
AUC: Area under the curve
TTD: Therapeutic target database
ROC: Receiver operating characteristic
