# Supplementary material for: SAveRUNNER: an R-based tool for drug repurposing
Source: BMC Bioinformatics. 2021 Mar 23;22:150. doi: 10.1186/s12859-021-04076-w (PMC7987121; doi:10.1186/s12859-021-04076-w)
Supplement: Supplementary file 3 — Additional file 3. Working example of SAveRUNNER application to COVID-19. [file 12859_2021_4076_MOESM3_ESM.pdf]

SAveRUNNER

Searching off-lAbel dRUg aNd NEtwoRk

GETTING STARTED

Software requirement

- SAveRUNNER has been developed in R (version 3.6.1) and tested on the following operative systems:
  - macOS High Sierra 10.13.6
  - Windows 10 Pro

Setting up

- Install R and R studio
- Download and unzip the SAveRUNNER software package (“*src*” folder)

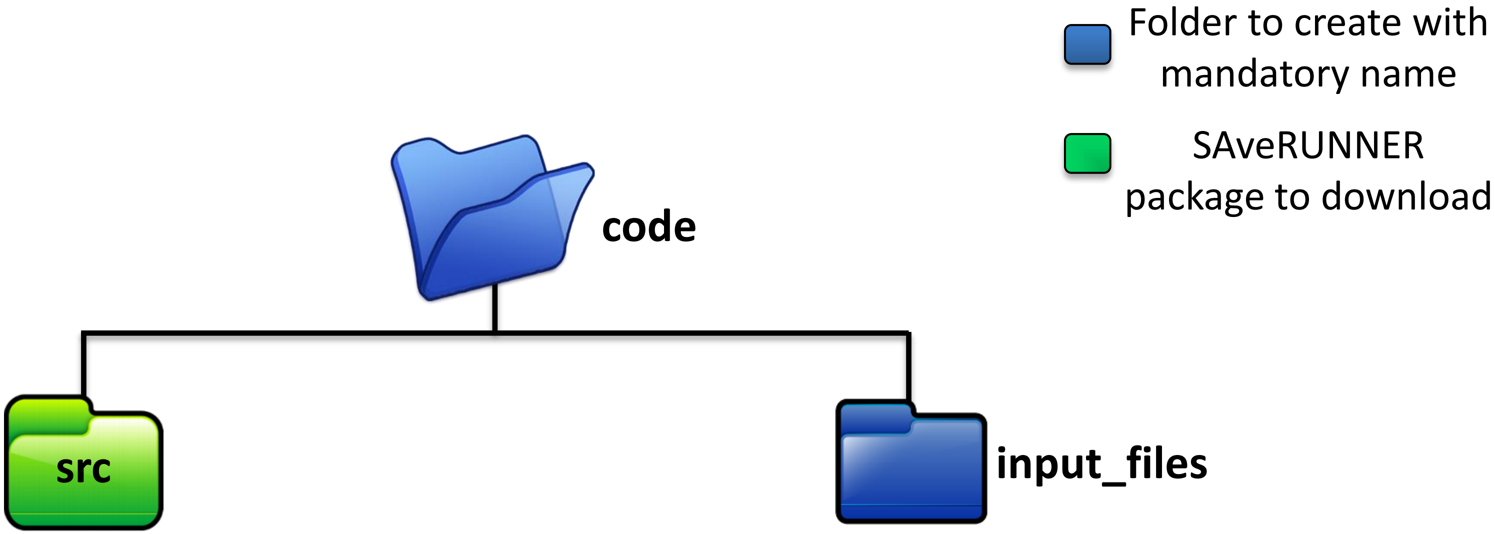
Folder to create with mandatory name
SAveRUNNER package to download
code
src
input_files

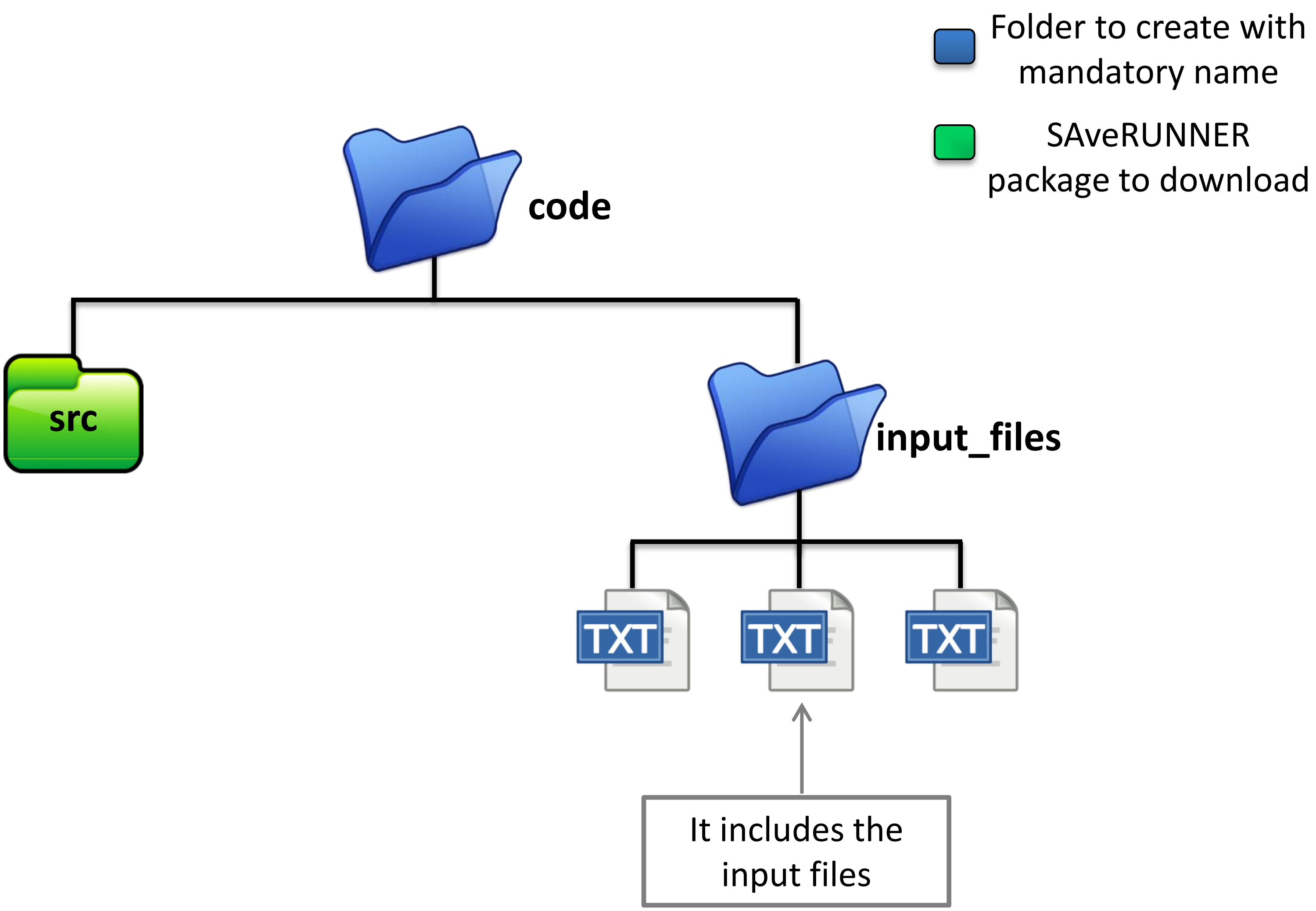
Folder to create with mandatory name
SAveRUNNER package to download
code
src
input_files
TXT
TXT
TXT
It includes the input files

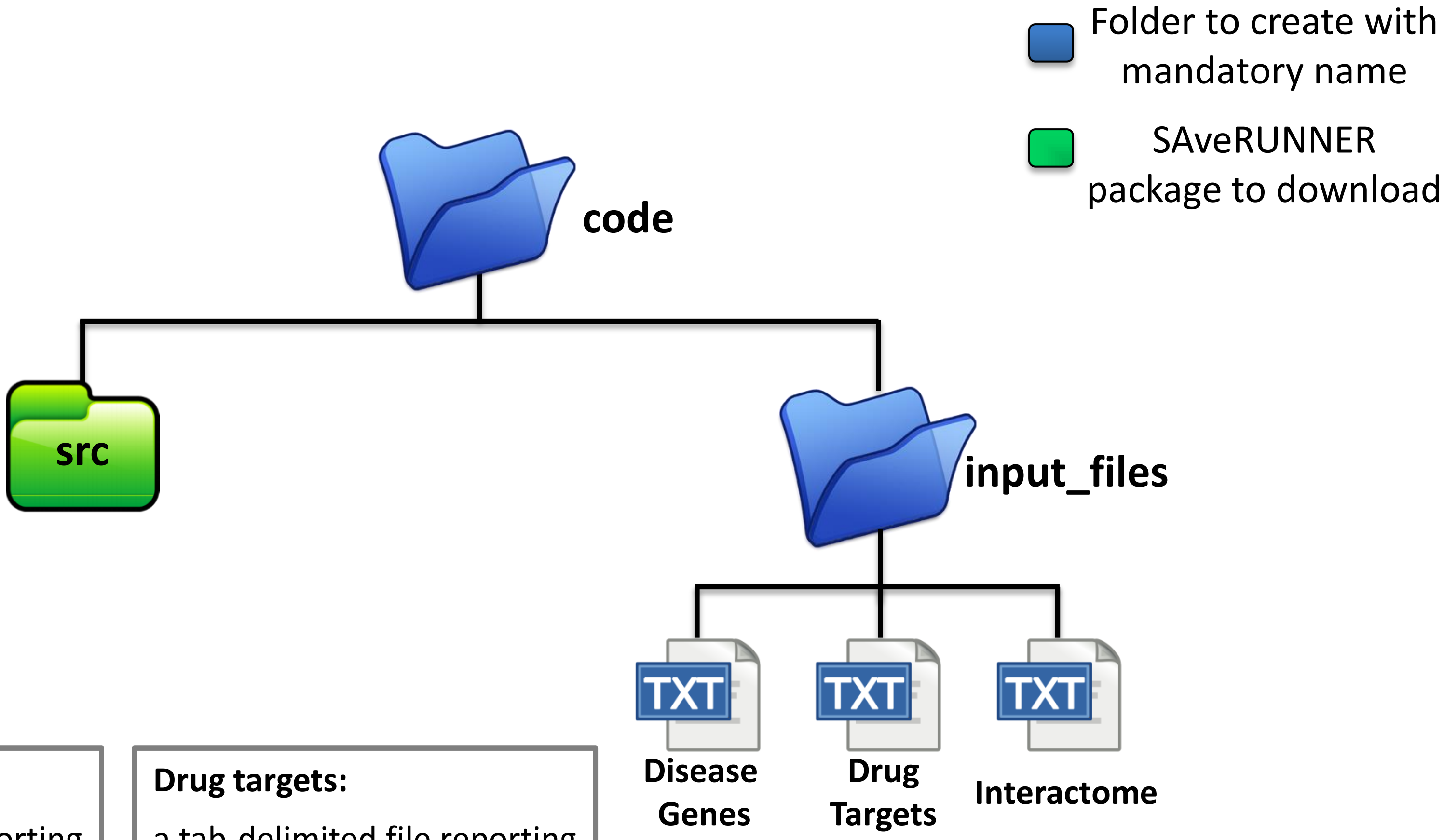

Disease genes:

a tab-delimited file reporting the disease-gene association as an edges list. It must include two columns: one named “**disease**” with the disease names and the other one named “**GeneID**” with the associated disease genes in Entrez Gene ID.

Drug targets:

a tab-delimited file reporting the drug-targets interactions as an edges list. It must include two columns: one named “**Drug**” with the drug names and the other one named “**GeneID**” with the interacting target genes in Entrez Gene ID.

Interactome:

a tab-delimited file reporting the human interactome. It must be provided as an edges list, i.e., it must include two columns reporting all the pairs of interacting genes in Entrez Gene ID.

Disease Genes - example

	A	B
1	disease	GeneID
2	COVID-19	25980
3	COVID-19	10157
4	COVID-19	26574
5	COVID-19	4363
6	COVID-19	28976
7	COVID-19	34
8	COVID-19	2181
9	COVID-19	8754
10	COVID-19	9510
11	COVID-19	8540
12	COVID-19	10270

Drug Targets - example

	A	B
1	Drug	GeneID
2	Zuclopenthixol	150
3	Zuclopenthixol	1813
4	Zuclopenthixol	1812
5	Zuclopenthixol	1816
6	Zuclopenthixol	3356
7	Zuclopenthixol	148
8	Zuclopenthixol	3269
9	Zucapsaicin	7442
10	Zotepine	1813
11	Zotepine	1812
12	Zotepine	1816
13	Zotepine	6530
14	Zotepine	3356
15	Zotepine	6532

Interactome - example

	A	B	C	D
1	Gene_A_Entrez ID	GeneB_Entrez_ID	data_source(s)	
2	1	310	IntAct,PINA	
3	1	368	IntAct,PINA	
4	1	1026	IntAct,PINA	
5	1	2886	IntAct,PINA	
6	1	3958	InnateDB	
7	1	4899	PINA	
8	1	6606	IntAct,PINA	
9	1	6622	IntAct,PINA	
10	1	7083	IntAct,PINA	
11	1	10321	HPRD,PINA	
12	1	10549	IntAct,PINA	
13	1	80854	IntAct,PINA	
14	2	60	IntAct,PINA	
15	2	250	IntAct	
16	2	259	BioGRID,HPRD,PINA	
17	2	292	IntAct	
18	2	293	IntAct	
19	2	309	HPRD	
20	2	310	IntAct,PINA	

source **target** **Db [optional]**

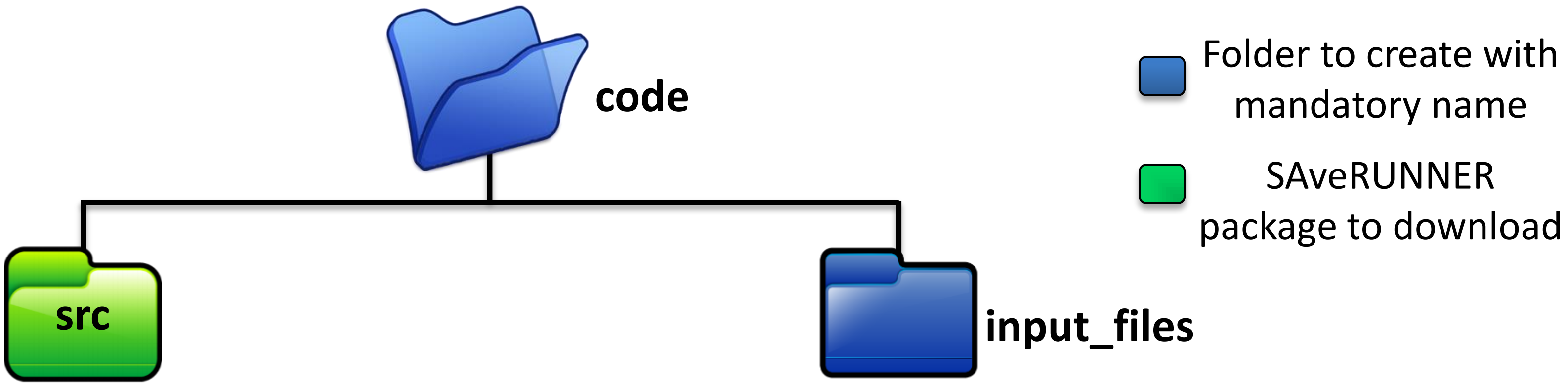
code
src
input_files
Folder to create with mandatory name
SAveRUNNER package to download

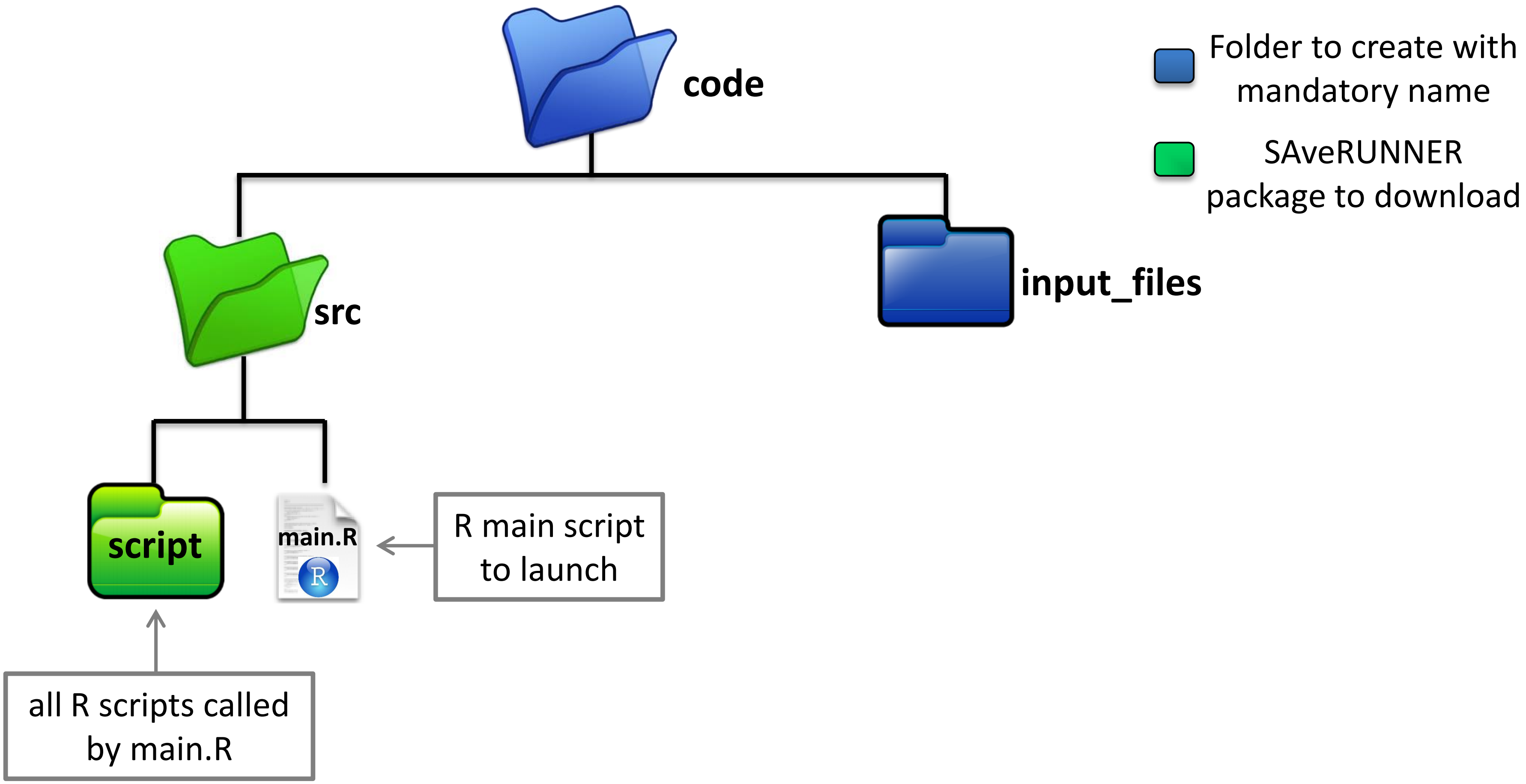

code
src
input_files
script
main.R
R main script to launch
all R scripts called by main.R
Folder to create with mandatory name
SAveRUNNER package to download

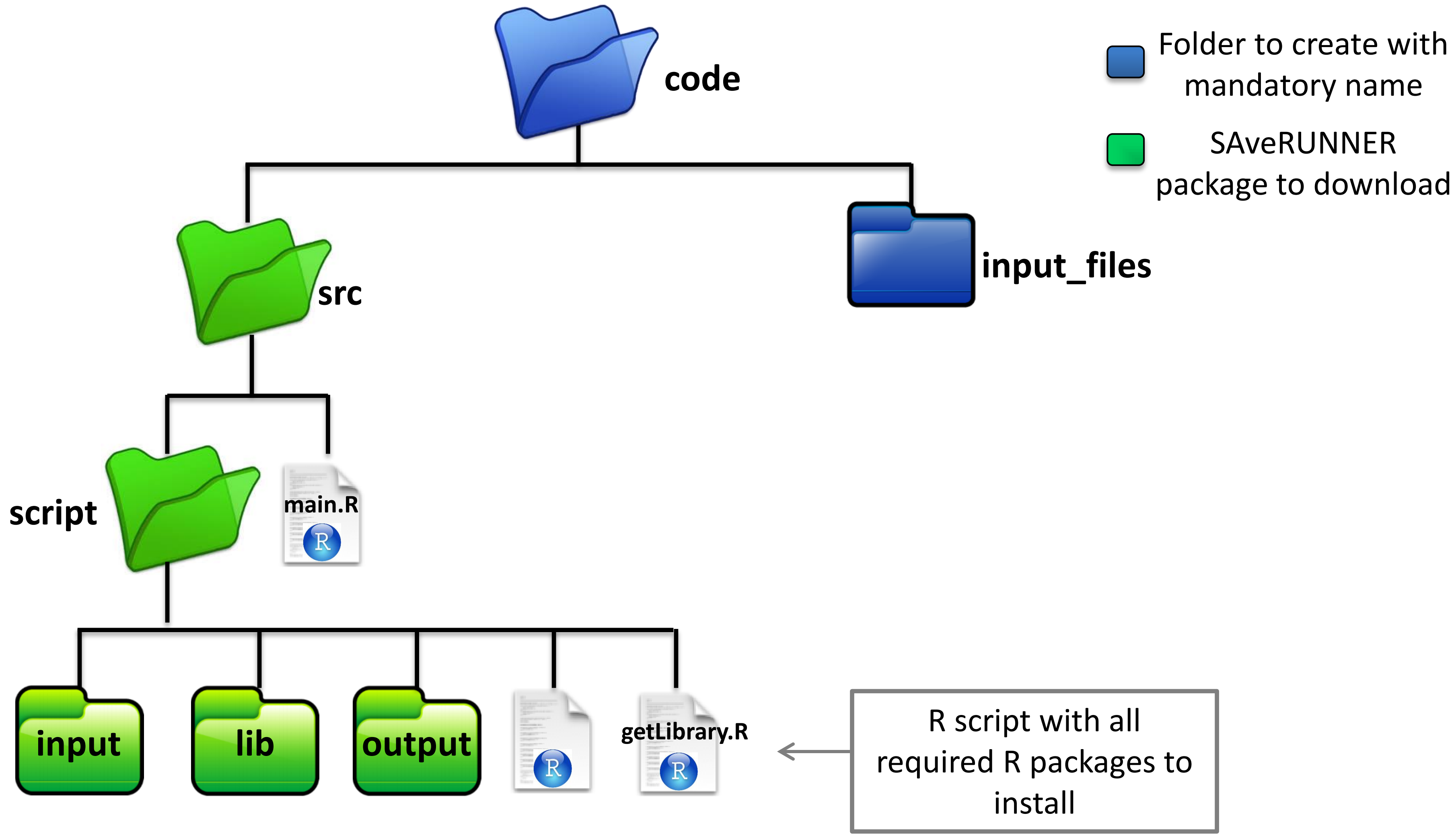

code
input_files
src
script
main.R
input
lib
output
getLibrary.R
R script with all required R packages to install
Folder to create with mandatory name
SAveRUNNER package to download

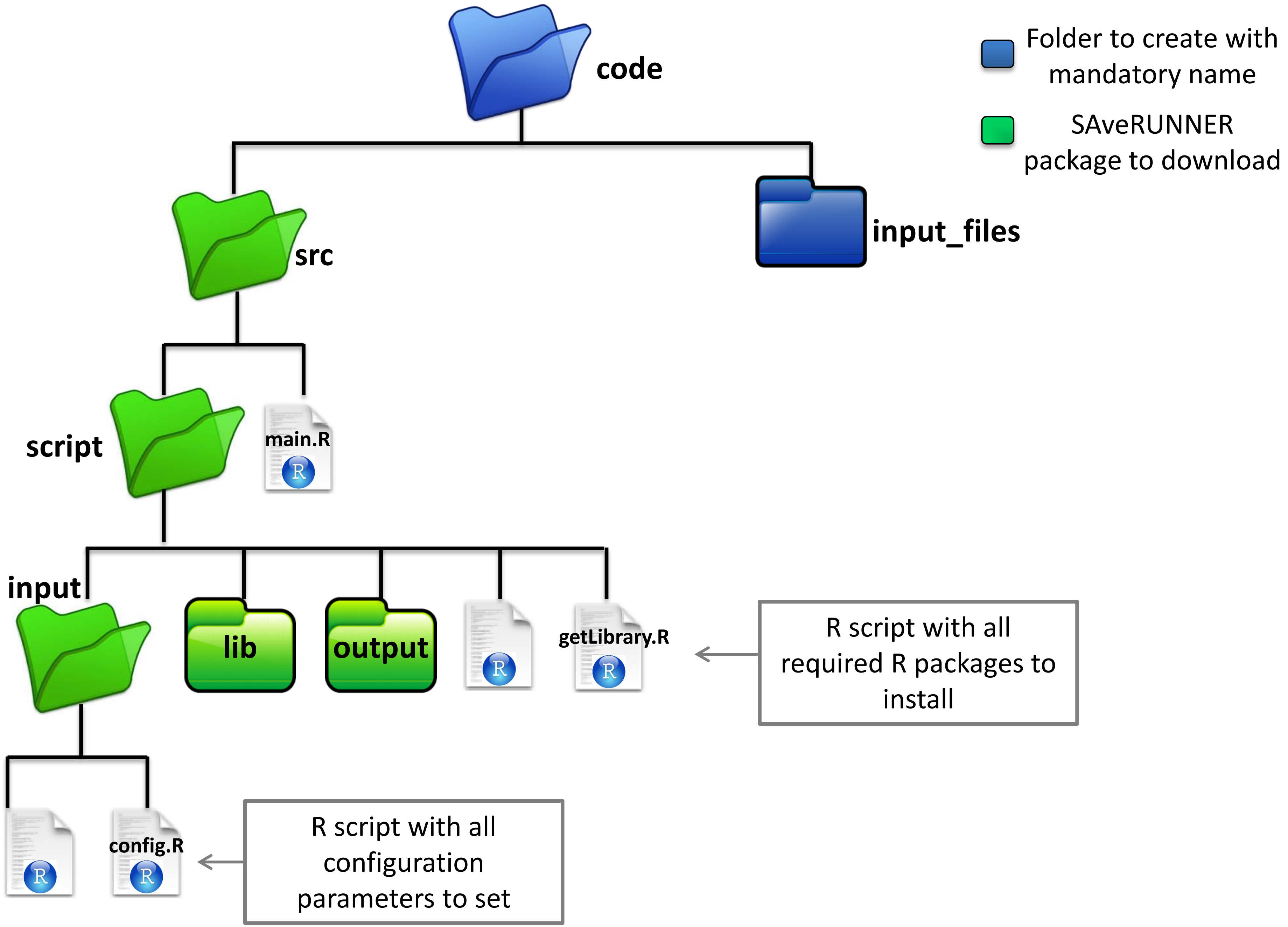

Folder to create with mandatory name
SAveRUNNER package to download
code
input_files
src
script
main.R
input
lib
output
getLibrary.R
R script with all required R packages to install
config.R
R script with all configuration parameters to set

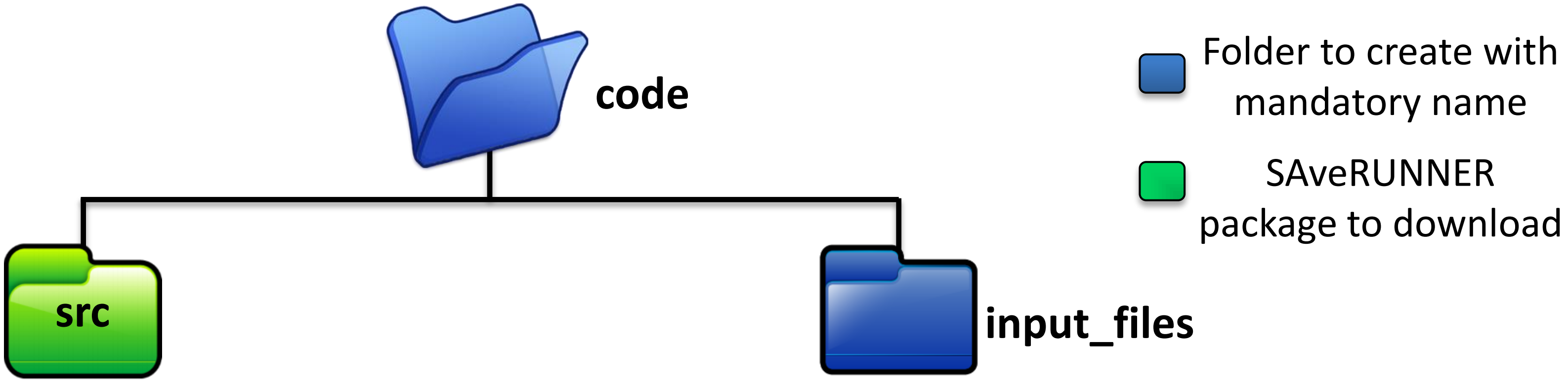
code
src
input_files
Folder to create with mandatory name
SAveRUNNER package to download

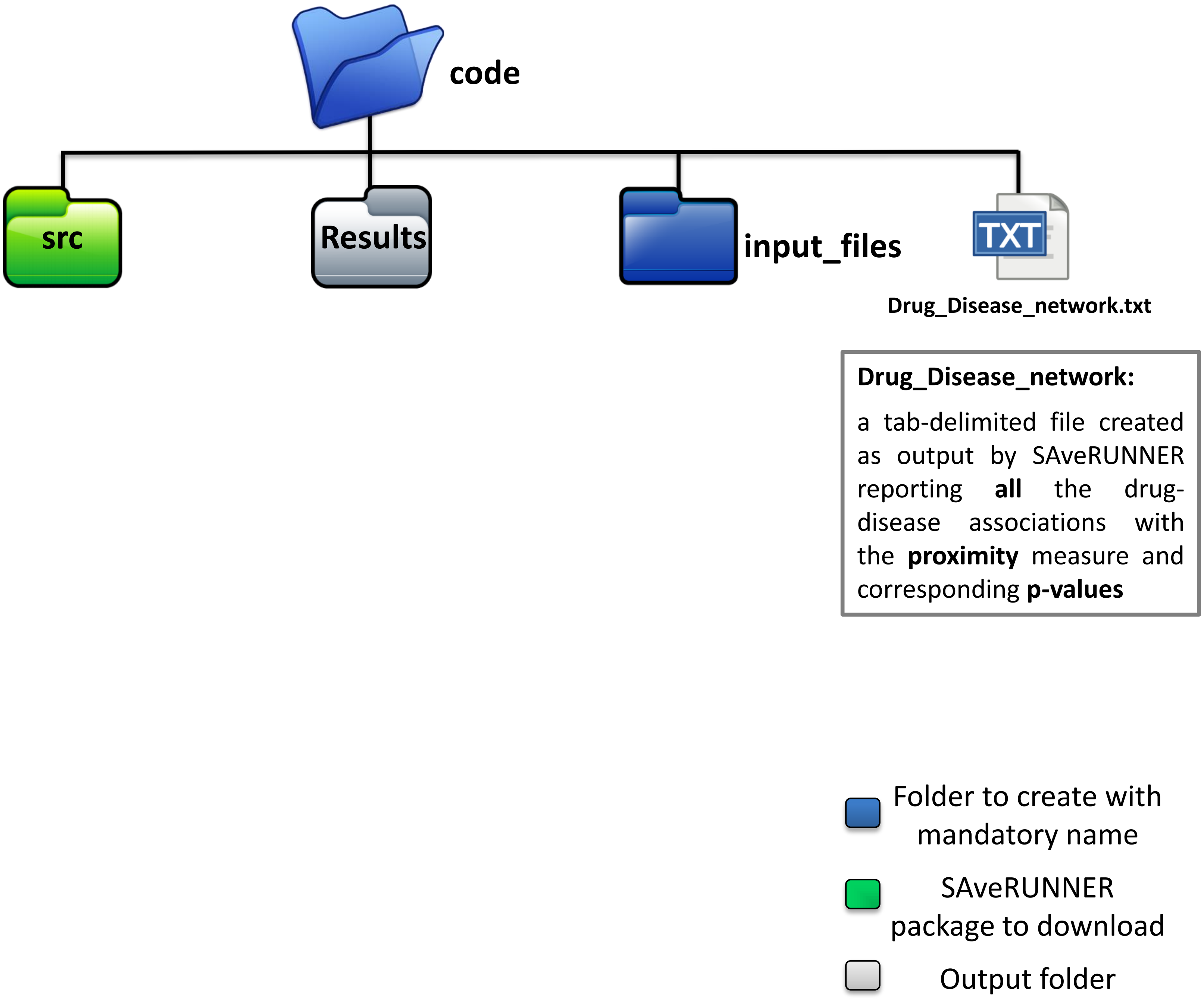
code
src
Results
input_files
TXT
Drug_Disease_network.txt
Drug_Disease_network:
a tab-delimited file created as output by SAveRUNNER reporting **all** the drug-disease associations with the **proximity** measure and corresponding **p-values**
Folder to create with mandatory name
SAveRUNNER package to download
Output folder

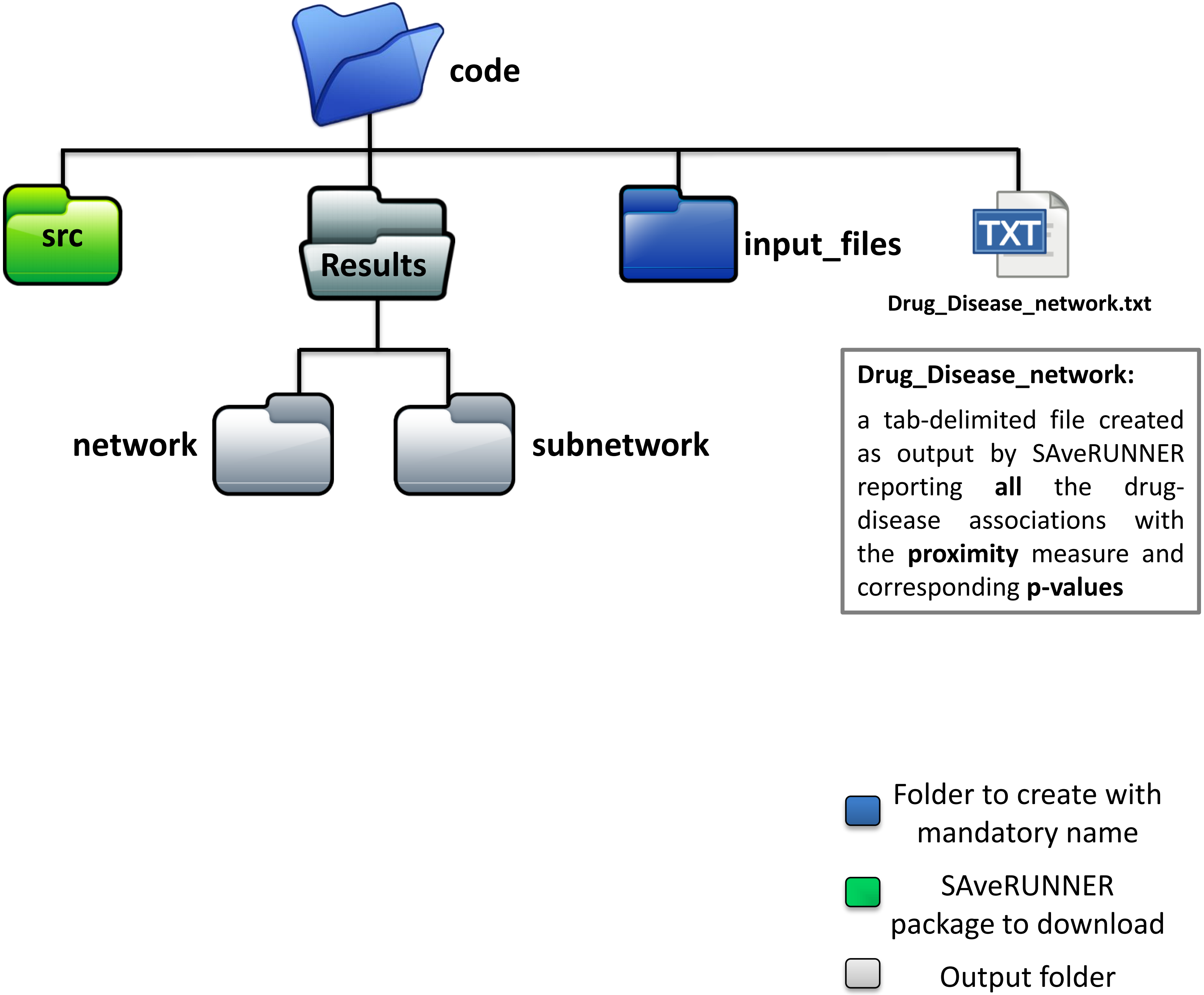
code
src
Results
input_files
TXT
Drug_Disease_network.txt
network
subnetwork
Drug_Disease_network:
a tab-delimited file created as output by SAveRUNNER reporting all the drug-disease associations with the proximity measure and corresponding p-values
Folder to create with mandatory name
SAveRUNNER package to download
Output folder

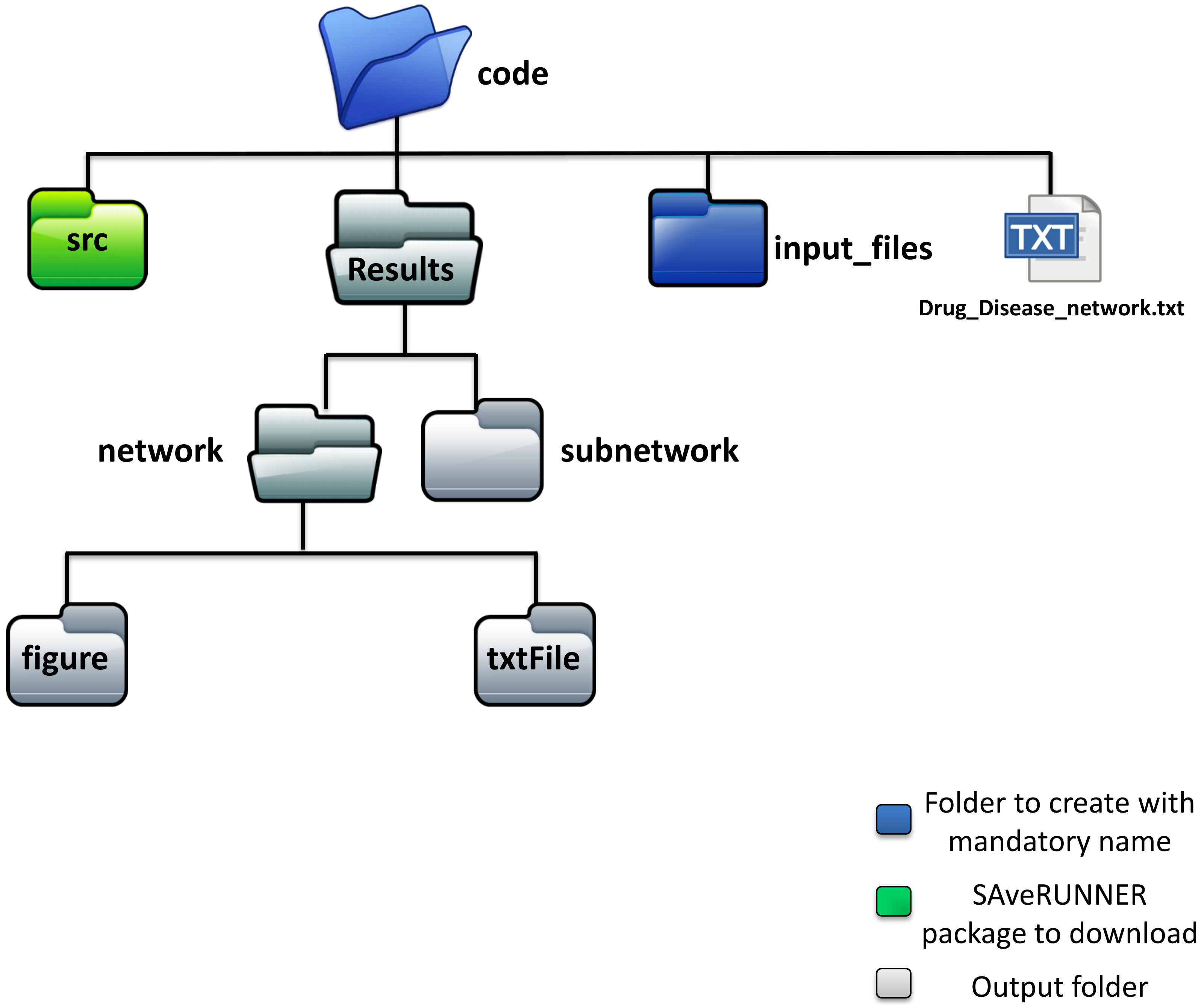
code
src
Results
input_files
TXT
Drug_Disease_network.txt
network
subnetwork
figure
txtFile
Folder to create with mandatory name
SAveRUNNER package to download
Output folder

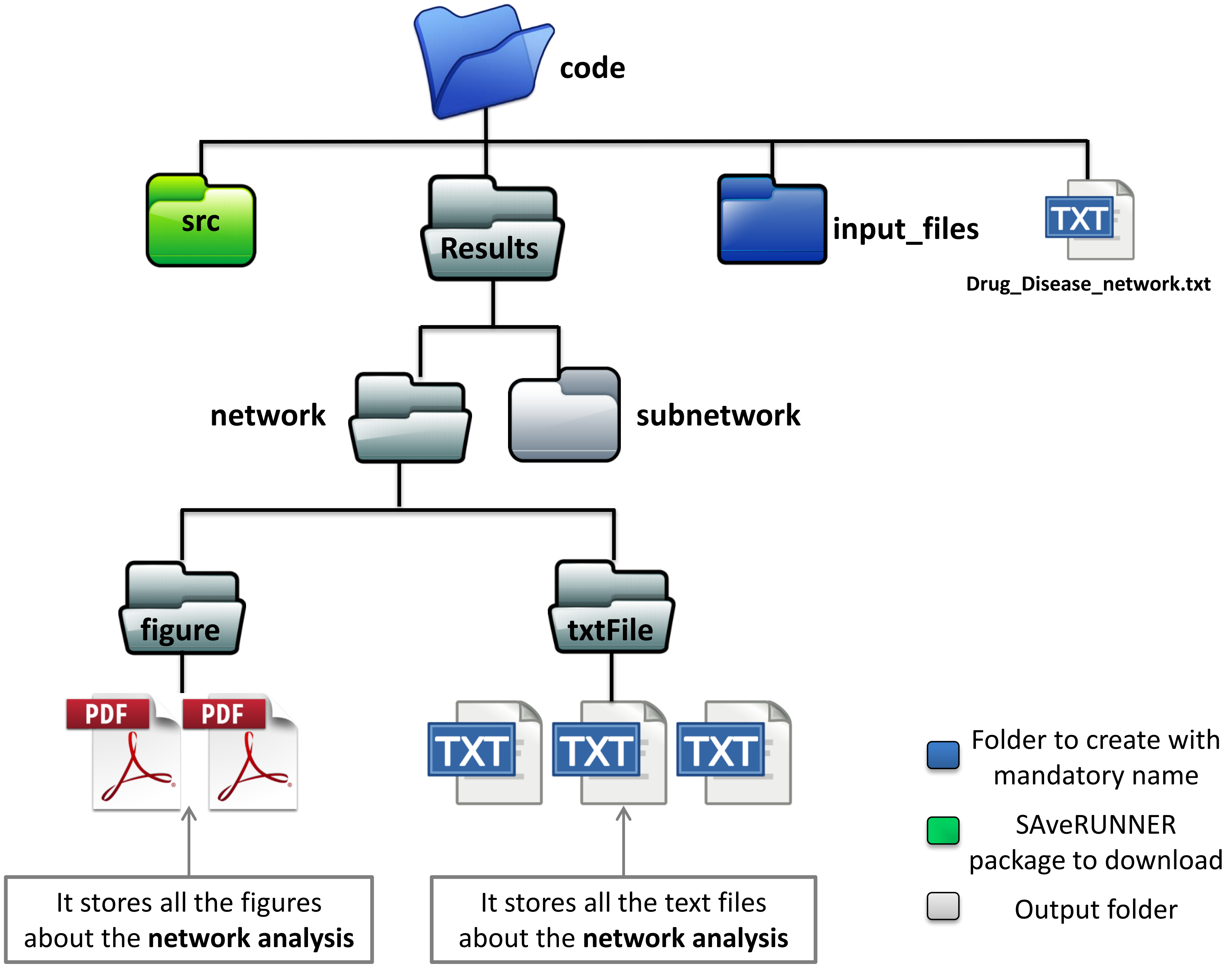
code
src
Results
input_files
TXT
Drug_Disease_network.txt
network
subnetwork
figure
txtFile
PDF
PDF
TXT
TXT
TXT
It stores all the figures about the **network analysis**
It stores all the text files about the **network analysis**
Folder to create with mandatory name
SAveRUNNER package to download
Output folder

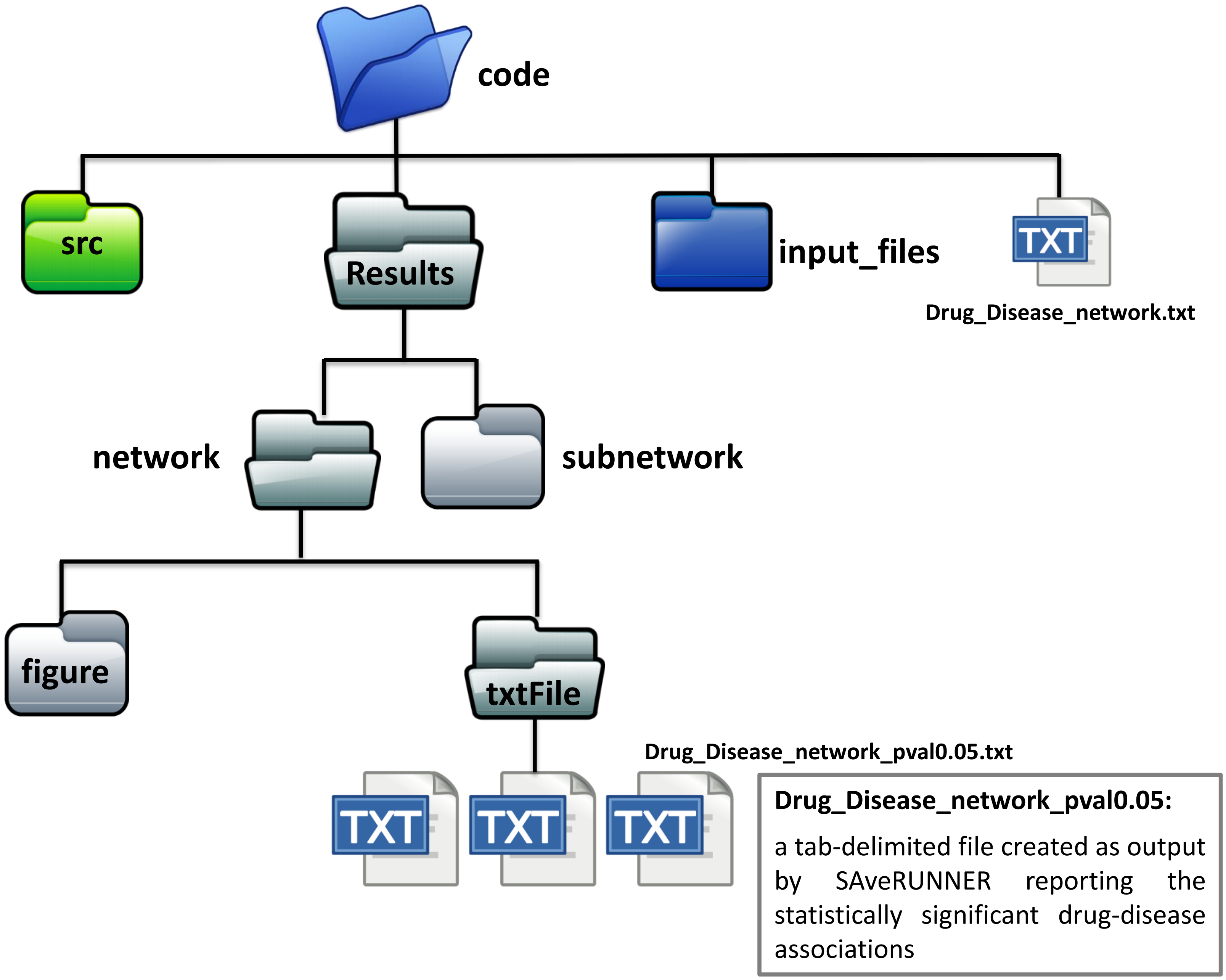
code
src
Results
input_files
TXT
Drug_Disease_network.txt
network
subnetwork
figure
txtFile
Drug_Disease_network_pval0.05.txt
TXT
TXT
TXT
Drug_Disease_network_pval0.05:
a tab-delimited file created as output by SAveRUNNER reporting the statistically significant drug-disease associations

Ready to launch!

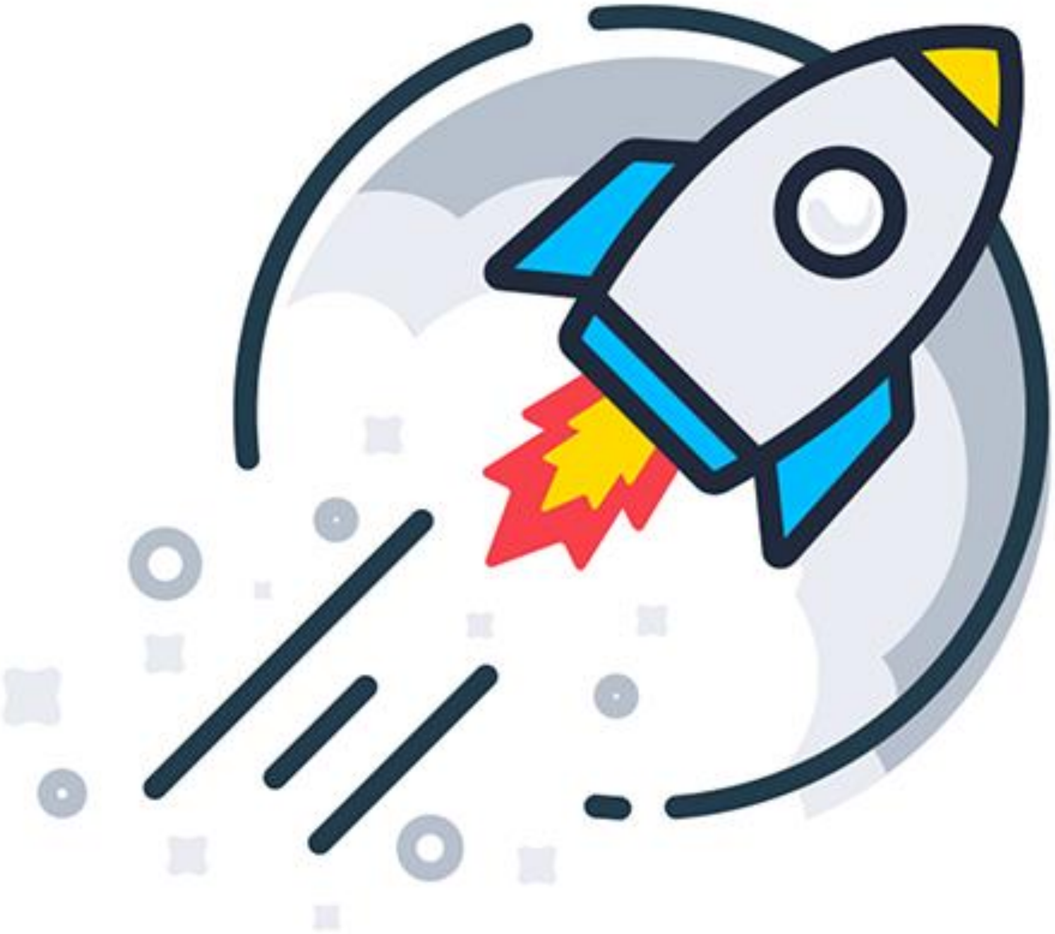

Main file

Initial settings

main.R

```r
rm(list=ls())

options(stringsAsFactors = F)

setwd("~/SaveRUNNER/Code/")
########################################
source("src/script/getLibrary.R")
source("src/script/getSource.R")
########################################
getLibrary()
getSource()
input_parameter <- config()
input_file <- inputFiles()
output_file <- networkFiles()
########################################
# compute drug-disease network
destfile = output_file$filename_out_allPval
if ( !file.exists(destfile) ) mainStartNetwork()

# select significative drug-disease association and (or not) adjust them
mainEndNetwork()
########################################
# make figure

if( length(input_parameter$diseases) > 1) mainFigure()
########################################
# create disease specific subnetwork
if( !is.null(input_parameter$sel_disease) ) mainSubnetwork()
########################################
```

Set the working directory

Main file

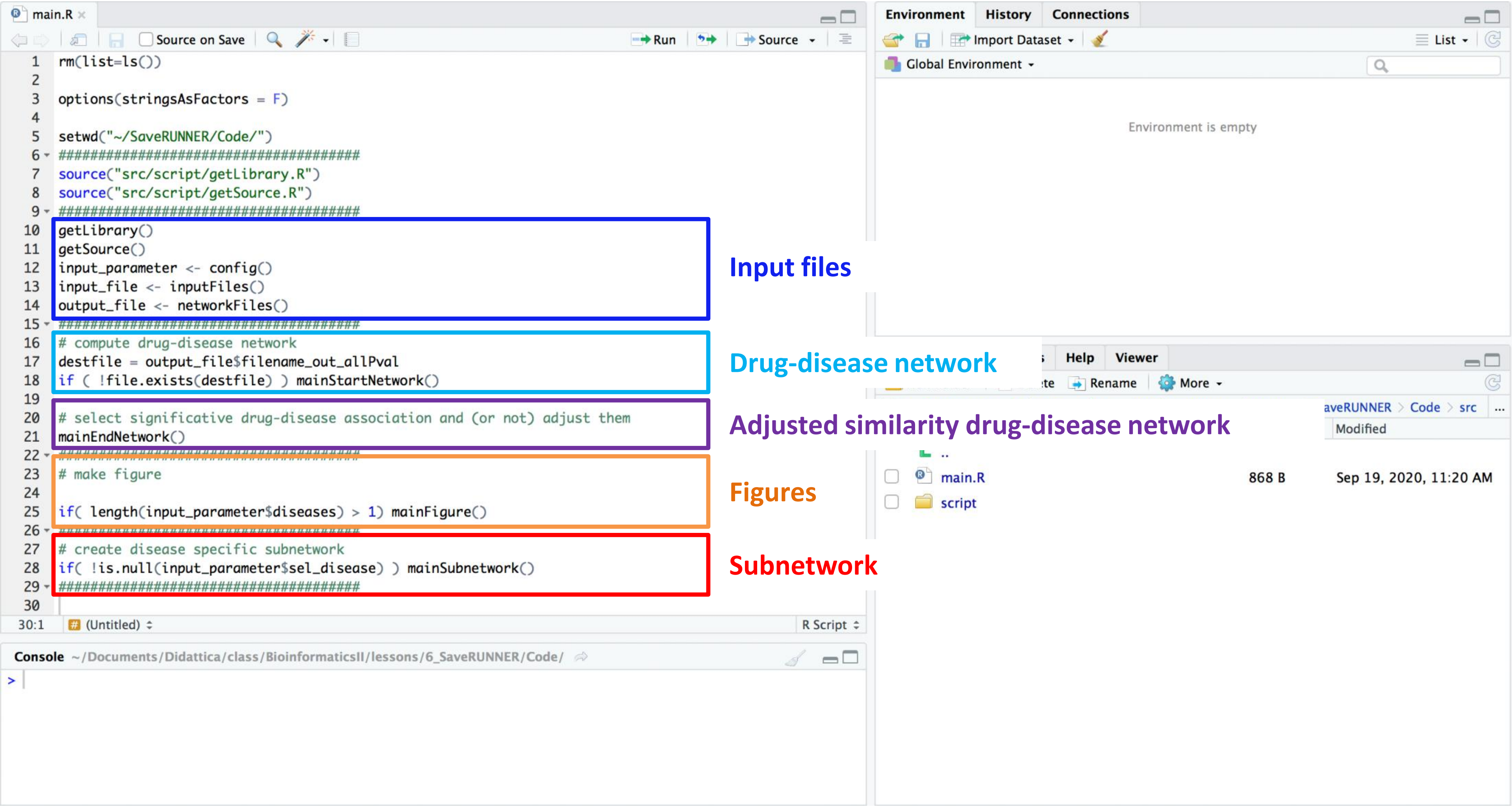

main.R
Source on Save
Run
Source
rm(list=ls())
options(stringsAsFactors = F)
setwd("~/SaveRUNNER/Code/")
##
source("src/script/getLibrary.R")
source("src/script/getSource.R")
##
getLibrary()
getSource()
input_parameter <- config()
input_file <- inputFiles()
output_file <- networkFiles()
##
# compute drug-disease network
destfile = output_file$filename_out_allPval
if (!file.exists(destfile)) mainStartNetwork()
# select significative drug-disease association and (or not) adjust them
mainEndNetwork()
##
# make figure
if(length(input_parameter$diseases) > 1) mainFigure()
##
# create disease specific subnetwork
if(!is.null(input_parameter$sel_disease)) mainSubnetwork()
##
Input files
Drug-disease network
Adjusted similarity drug-disease network
Figures
Subnetwork
30:1
(Untitled)
R Script
Console ~/Documents/Didattica/class/BioinformaticsII/lessons/6_SaveRUNNER/Code/
Environment
History
Connections
Import Dataset
List
Global Environment
Environment is empty
Help
Viewer
Rename
More
aveRUNNER > Code > src
Modified
main.R
868 B
Sep 19, 2020, 11:20 AM
script

Main file

Initial settings

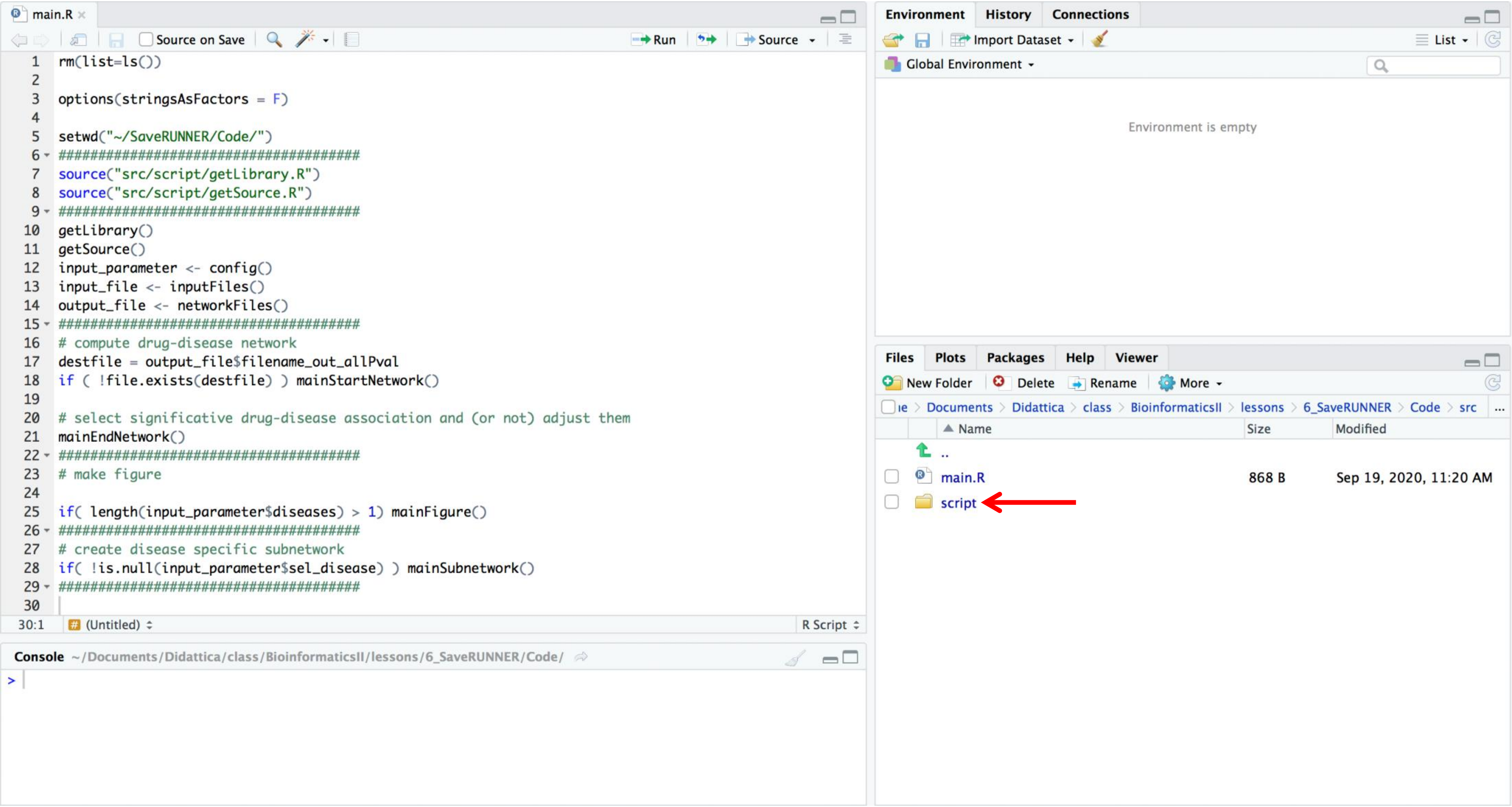

Main file

Initial settings

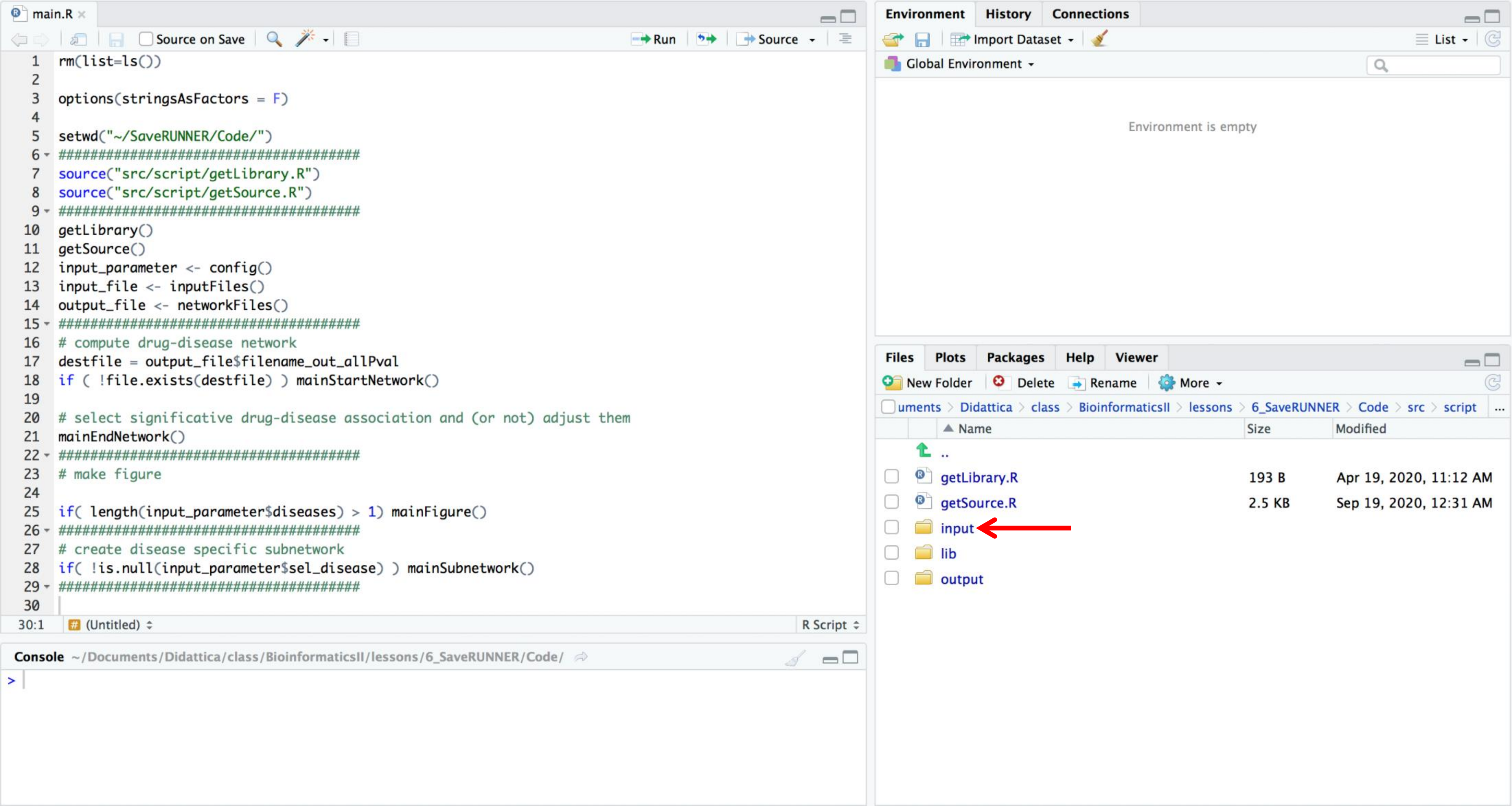

Main file

Initial settings

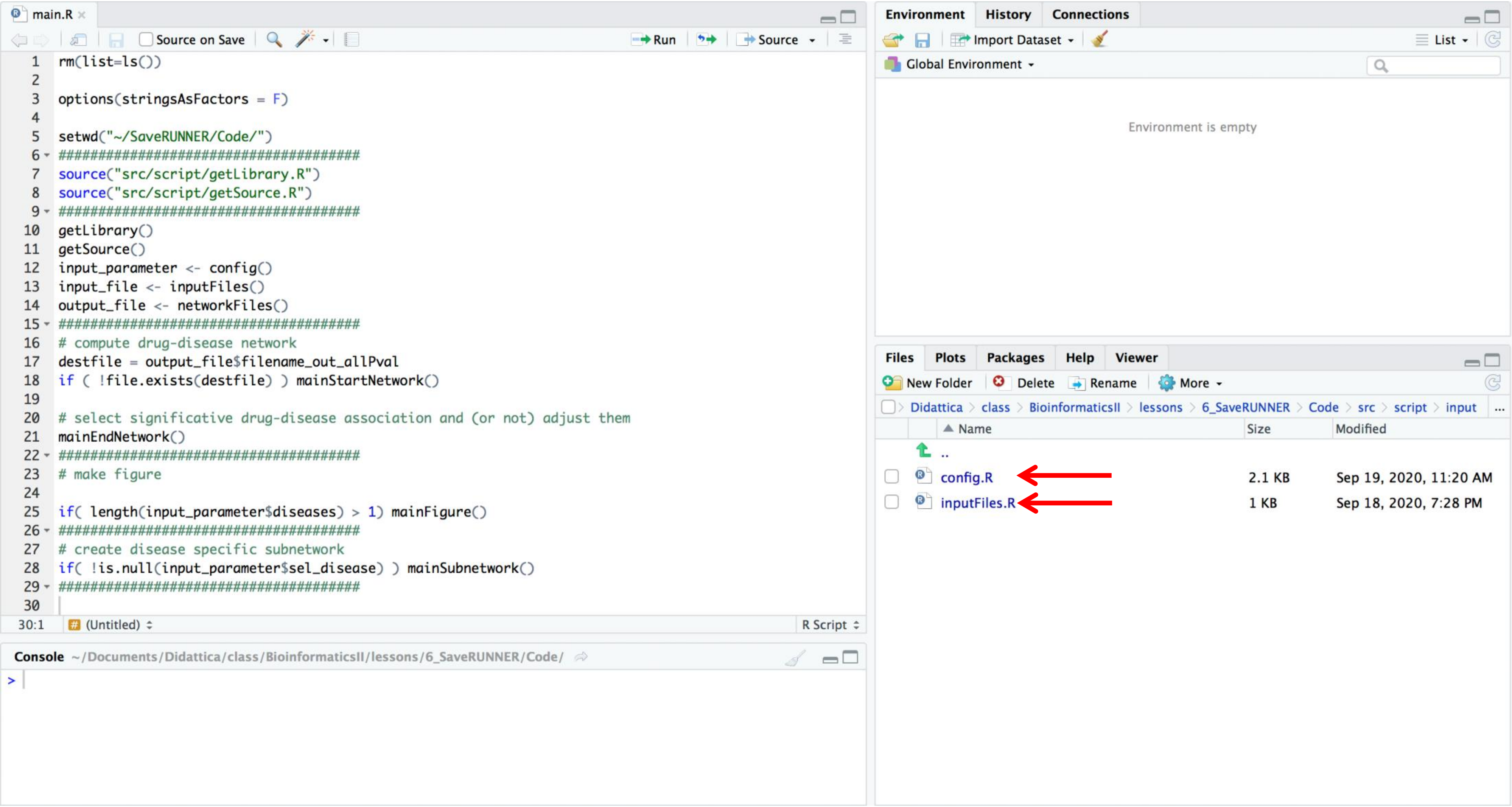

Configuration file

Initial settings

```r
config <- function(){

  # for executing SAveRUNER launch:
  # source('~/SaveRUNNER/code/src/main.R')
  ##################################
  # parameters for computing start network with edge-weight =

  diseases <- "COVID-19"
  ##################################
  # parameters for computing end network

  dirRes <- "Results/"

  interaction = "similarity"  # edge-weight = similarity or proximity
  pval_thr = 0.05             # select significative drug-disease association
  adjust_link = F            # adjust similarity or not
  new_link = F                # add new drug-disease association or not (without compute pval)
  ##################################
  # parameters for making figure

  if( (interaction == "proximity") ) distance = "proximity"
  if( (interaction == "similarity") & (adjust_link == F) ) distance = "similarity"
  if( (interaction == "similarity") & (adjust_link == T) ) distance = "adjusted_similarity"
  ##################################
  # parameters for computing subnetwork

  sel_drug = NULL
  sel_disease = NULL
  ##################################
```

Short command line to launch SAveRUNNER

Environment is empty

Name	Size	Modified
config.R	1.6 KB	Jan 27, 2021, 12:58 PM
inputFiles.R	1.1 KB	Dec 21, 2020, 10:21 AM

Configuration file

Initial settings

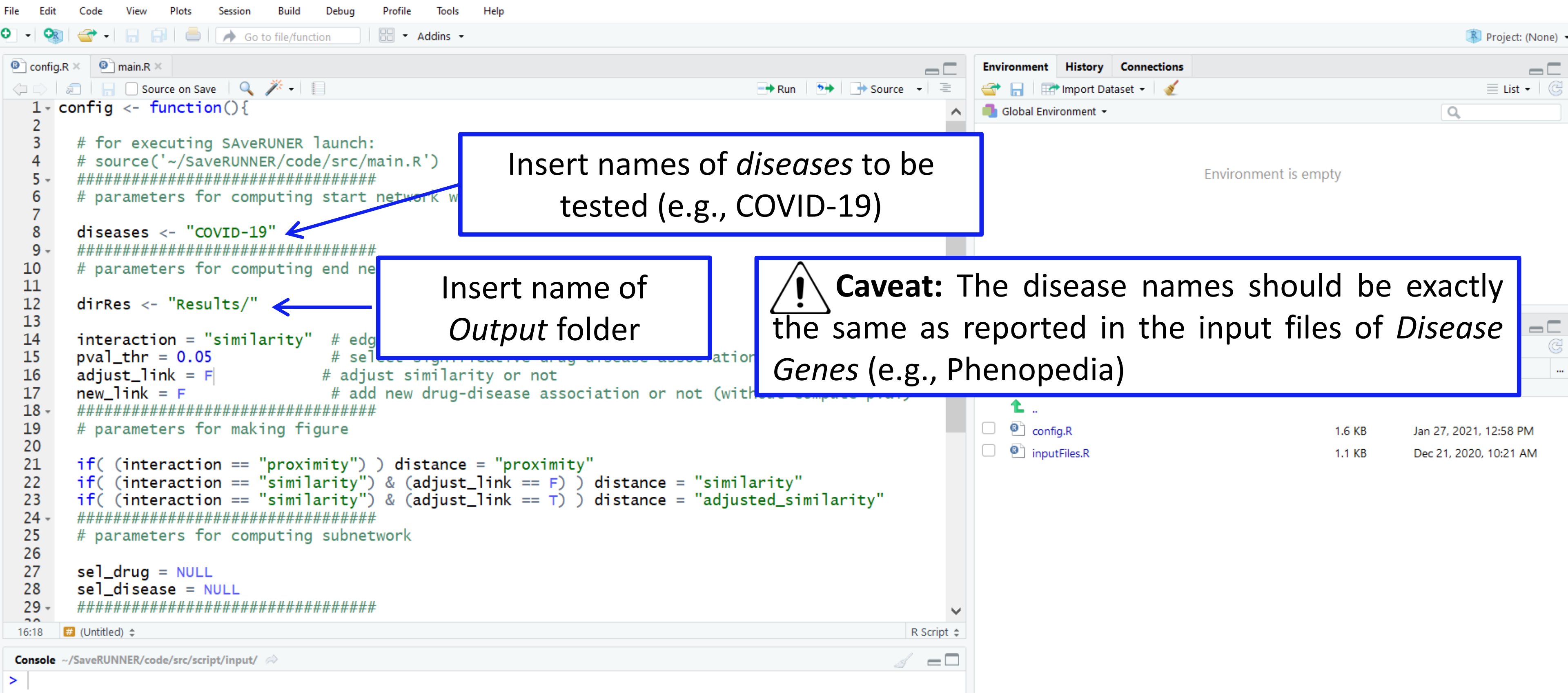

Configuration file

Initial settings

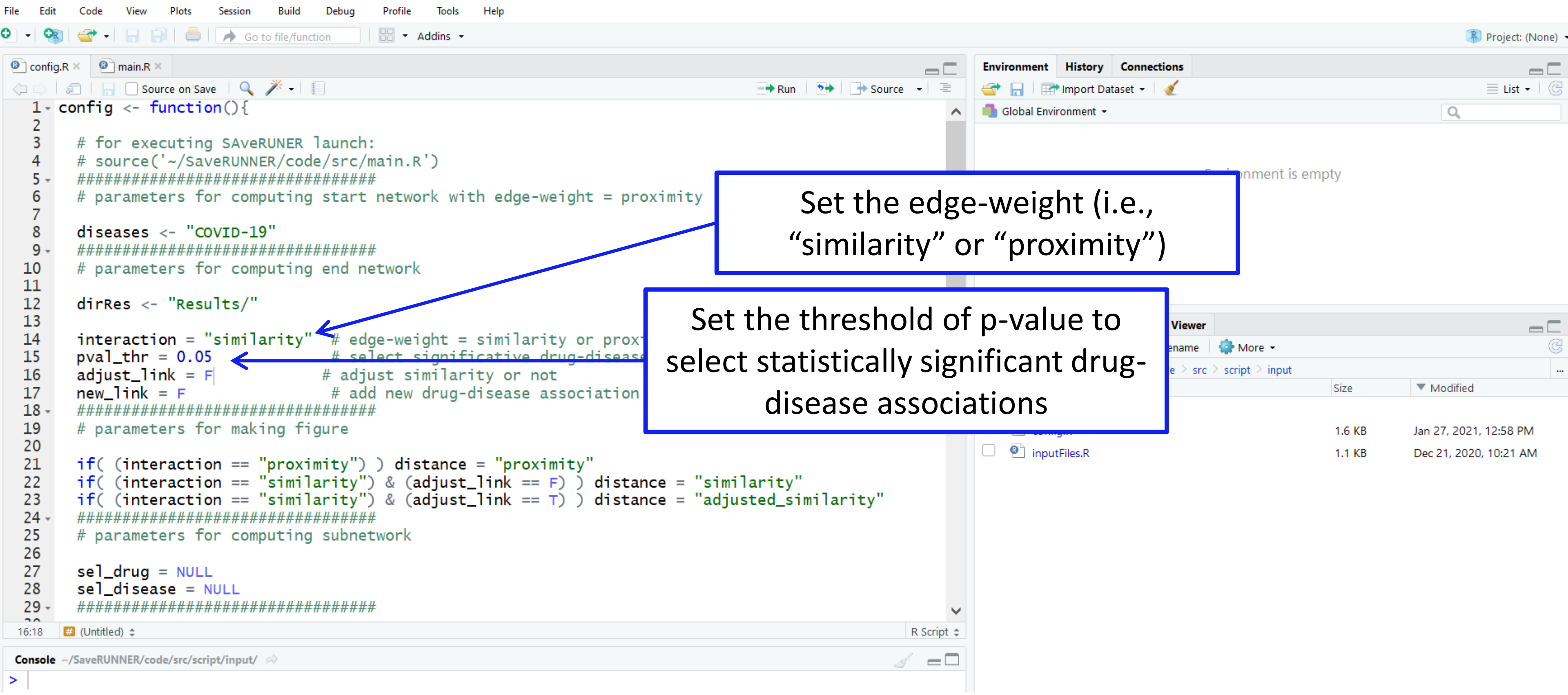

Network-based drug disease similarity

- The **similarity measure** between drug module and disease module is defined as follows:

$$similarity = \frac{\max(p) - p}{\max(p)}$$

- where p is the network proximity measure
- It assumes values ranging in [0,1]

Null similarity means that the corresponding disease and drug modules are **very distal** in the human interactome (i.e., p is maximum); whereas **maximum similarity** means that the corresponding disease and drug modules are **very proximal** in the human interactome (i.e., p equal to zero).

- The **proximity** between the lists of drug targets and disease genes is defined as:

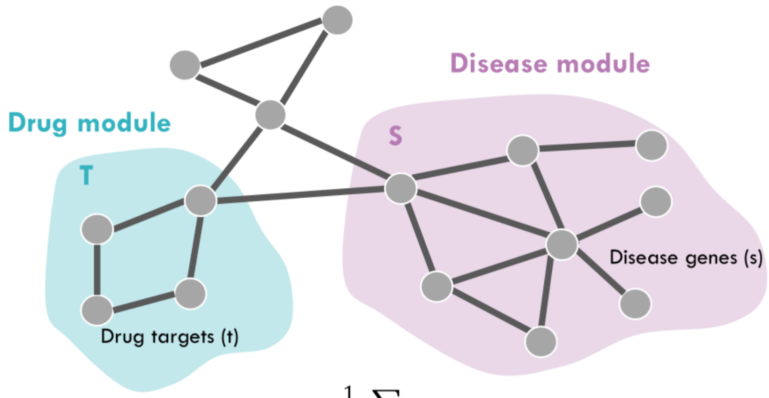

$$p(T,S) = \frac{1}{\|T\|}\sum_{t \in T} \min_{s \in S} d(t,s)$$

T = set of drug targets
S = set of disease genes
p = average shortest path length between drug targets t in the drug module T and the nearest disease genes s in the disease module S

Output

- The **similarity measure** between drug module and disease module is defined as follows:

$$similarity = \frac{\max(p) - p}{\max(p)}$$

- where p is the network proximity measure
- It assumes values ranging in [0,1]

Null similarity means that the corresponding disease and drug modules are **very distal** in the human interactome (i.e., *p* is maximum); whereas **maximum similarity** means that the corresponding disease and drug modules are **very proximal** in the human interactome (i.e., *p* equal to zero).

- **Statistically significant drug-disease associations** are selected according to the significance level for the p-values (default: p-value ≤ 0.05)
- It returns the **proximity and/or similarity** drug-disease network

disease	drug	proximity	pval	similarity
COVID-19	acrivastine	1.00000000	4.220080e-02	0.8000000
COVID-19	alcaftadine	1.00000000	4.161242e-02	0.8000000
COVID-19	alimemazine	1.00000000	4.484301e-02	0.8000000
COVID-19	alverine	1.00000000	4.578700e-02	0.8000000
COVID-19	amifampridine	1.00000000	3.433878e-02	0.8000000
COVID-19	aminocaproic acid	0.66666667	3.054591e-03	0.8666667
COVID-19	amrinone	1.00000000	4.953049e-03	0.8000000
[illegible]	[illegible]	[illegible]	[illegible]	[illegible]
[illegible]	[illegible]	[illegible]	[illegible]	[illegible]
COVID-19	antihemophilic factor human	1.00000000	3.700048e-02	0.8000000
COVID-19	asparagine	1.16666667	1.088828e-02	0.7666667
COVID-19	astemizole	1.00000000	2.194849e-02	0.8000000
COVID-19	avanafil	1.00000000	5.180000e-06	0.8000000
COVID-19	azacitidine	0.00000000	6.440000e-08	1.0000000
COVID-19	azatadine	1.00000000	3.414587e-02	0.8000000
COVID-19	bacitracin	0.50000000	3.304639e-03	0.9000000
COVID-19	bepotastine	1.00000000	1.670887e-02	0.8000000

Drug_Disease_network_pval0.05.txt

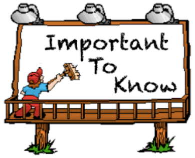

Given a disease A and a drug b, if the **p-value** associated to their distance in the human interactome is smaller of the chosen significance level (default 0.05), the probability that the off-label drug b would be effective for this disease A is greater than expected by chance
