# Supplementary material for: SAveRUNNER: an R-based tool for drug repurposing
Source: BMC Bioinformatics. 2021 Mar 23;22:150. doi: 10.1186/s12859-021-04076-w (PMC7987121; doi:10.1186/s12859-021-04076-w)
Supplement: Supplementary file 4 — Additional file 4. Implementation of BiRW-based algorithm for drug repurposing. [file 12859_2021_4076_MOESM4_ESM.pdf]

Bi-Random walk-based algorithm

Inspired by an already existing algorithm of drug repurposing called MBiRW [1], we implemented a Bi-Random walk-based (BiRW) approach to infer potential reuse for existing drugs. We started from the known drug–disease associations available on the TTD [2] and iteratively we added new associations integrating information retrieved from DrugBank [3], for what concerns targets of already approved drugs, and from Phenopedia [2], for what concerns disease genes. Denoting with m the total number of the approved drugs and n the total number of diseases, the known drug-disease associations were translated into a binary matrix $W_{rd}^{m \times n}$ by assigning 1 if the given drug (matrix row) is associated to a given disease (matrix column), and 0 otherwise. Next, a weighted adjacency matrix $W_{rr}^{m \times m}$ was computed to model drug similarity, where weights are the number of common targets between any pair of drugs. Further, a weighted adjacency matrix $W_{dd}^{n \times n}$ was computed to model disease similarity, where weights are the number of common disease genes between any pair of diseases (Figure S1 a).

In analogy with [1], the matrices W_{rr} and W_{dd} were adjusted based on the known drug–disease associations. The underlying hypothesis is that drug (disease) pairs, whose similarity value is greater of what expected by chance, are more likely to share common diseases (drugs).

From the adjusted matrix W_{rr}, a weighted *drug similarity network* was built, in which nodes are the approved drugs and an edge occurs between two nodes if they share at least one target gene, with a weight given by the corresponding element of the matrix W_{rr}. Likewise, from the adjusted matrix W_{dd}, a weighted *disease similarity network* was built, in which nodes are the diseases, and an edge occurs between two diseases if they share at least one disease gene, with a weight given by the corresponding element of the matrix W_{dd}.

Next, new drug–disease associations were predicted through an iterative random walk process on drug and disease similarity networks, simultaneously. Denoting with the parameters l and r the walks in the drug network (left walks) and in the disease network (right walks), the bi-random walk can be described by the following equations:

$$A_t^l = \alpha W_{rr} A_{t-1} + (1 - \alpha) A_0$$

$$A_t^r = \alpha A_{t-1} W_{dd} + (1 - \alpha) A_0$$

where A_0 denotes the drug-disease association at $t = 0$ (matrix W_{rd}), while A_t^l and A_t^r represent the predicted drug–disease associations at iteration t starting from left or right, respectively. The hypothesis behind is that an association between a drug R1 and a disease D1 can be added following these two avenues (Figure S1 b):

1. a random walker starts from a random vertex R1 of the drug similarity network and in each step walks to one of the neighboring vertices (for instance R2, with a known or previously predicted association with D1) with a probability proportional to the weight of the edge traversed (i.e., the corresponding element of W_{rr})
2. a random walker starts from a random vertex D1 of the disease similarity network and in each step walks to one of the neighboring vertices (for instance D2, with a known or previously predicted association with R1) with a probability proportional to the weight of the edge traversed (i.e., the corresponding element of W_{dd})

In both cases, the existence of a known association between a drug R1 and a disease D1 has been considered with a probability $(1 - \alpha)$. Then, in each step of the iteration process, the predicted drug–disease associations matrix A_t is given by the mean between A_t^l and A_t^r.

Finally, a bipartite drug-disease network was constructed consisting of two sets of nodes: one set corresponding to all disease, the other set corresponding to all approved drugs. An edge between a drug and a disease occurs if an association is already known or has been predicted among them.

Tenfold cross-validation

The performance of BiRW-based algorithm in predicting new drug–disease associations was evaluated through a tenfold cross validation [1]. This is a technique to investigate the predictive power of an algorithm by partitioning the original sample into a training and test set. In particular, the tenfold cross-validation consists of randomly partitioning the original sample (in our case the drug-disease associations) into 10 equal size subsamples. Of the 10 subsamples, a single subsample is retained as test set and the remaining 9 subsamples

are used as training set. The cross-validation process is then repeated for 10 iterations so that each of the 10 subsamples is used exactly once as the test set.

The results of each iteration were evaluated in terms of ROC probability curves. In particular, each time, the predicted drug-disease associations were ranked according to the similarity score estimated by the BiRW-based algorithm (i.e., the corresponding element of matrix A_t) and the N top-ranked drug-disease associations were selected to be evaluated by the ROC curve analysis, where N is the length of the test set. It's worth to stress that, in each cross-validation trial, we didn't use the information about the known drug–disease associations for the test set that were put to zero at first iteration of the bi-random walk process. Then, the ROC curve is constructed for different values of a specified threshold, where a true drug–disease association was considered as correctly predicted if the estimated similarity score of this association was higher than the specified threshold. The results of the ROC curve analysis obtained for each iteration were averaged to obtain a mean ROC curve and the corresponding Area Under the Curve (AUC) was calculated. Higher the AUC, better the algorithm is at distinguishing between two classes (i.e., known drug-disease associations *versus* unknown drug-disease associations).

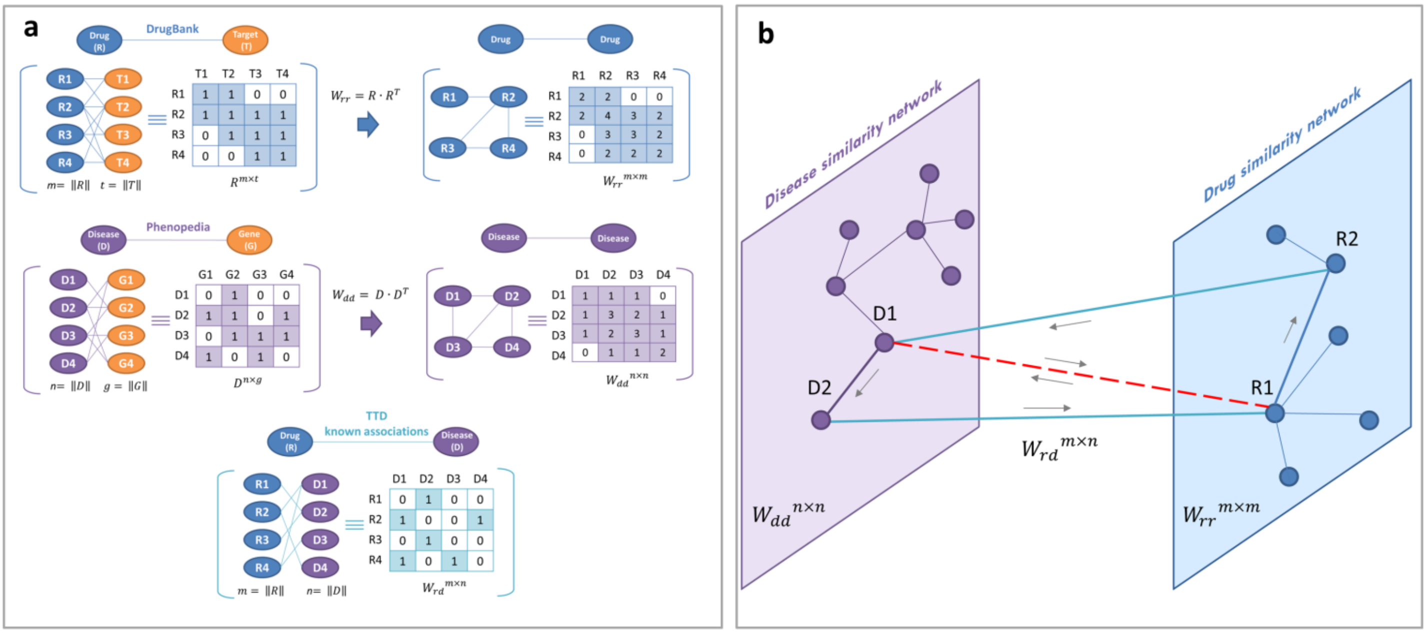

Figure S1. BiRW-based algorithm. Schematic representation of the construction of input matrices (a) and predicted drug-disease associations network (b).

Supplementary references

1. Luo, H. *et al.* Drug repositioning based on comprehensive similarity measures and Bi-Random walk algorithm. *Bioinformatics* **32**, 2664–2671 (2016).
2. Yu, W., Clyne, M., Khoury, M. J. & Gwinn, M. Phenopedia and Genopedia: disease-centered and gene-centered views of the evolving knowledge of human genetic associations. *Bioinformatics* **26**, 145–146 (2010).
3. Wishart, D. S. *et al.* DrugBank 5.0: a major update to the DrugBank database for 2018. *Nucleic Acids Res.* **46**, D1074 (2018).
